# Development of adaptive activation functions with curvature and range modulation for abstract image feature learning in complex CNNs for cross-domain applications

**DOI:** 10.1371/journal.pone.0355613

**Published:** 2026-08-14

**Authors:** Ali Raza, Akhtar Ali, Sami Ullah, Basit Rehman, Zafar Ali

**Affiliations:** 1 Department of Mathematics, Government College University, Faisalabad, Pakistan; 2 Department of Computer Science, Government College University, Faisalabad, Pakistan; 3 Department of Mathematics, Brunel University, London, United Kingdom; University of Engineering and Technology Taxila Pakistan, PAKISTAN

## Abstract

Deep learning architectures comprise hierarchies in modern machine learning studies, and these are not only full of semantic depth; they are also structured systematically by a sequence of nonlinear transformations. In particular, in convolutional neural networks, the activation functions are the canonical nonlinear construction blocks that control the expressiveness of the model, gradient flow in the process of back-propagation, and hierarchical feature abstraction. The canonical activation functions, which are the rectified linear unit (ReLU), the sigmoid, and the hyperbolic tangent (tanh) have non-adaptive and non-dynamic operation properties. As a result, they limit the capacity of a network to learn the complex nonlinear dynamics of a variety of data distributions and maximize the multi-level dependencies that occur among hierarchical layers. To overcome the above constraints, the current proposal proposes the implementation of **Symmetric Modifiable Slope Activation (SMSA)**, which is a method that enables the dynamically calibrated neural layers through simultaneous modification of the slope, rang and the curvature of the activation space. SMSA is implemented in three different forms, first is slope parameter (*n*) is defined manually, a globally adjustable one with a learnable coefficient β, and (iii) a layer-wise adaptive regime allowing each convolutional layer to learn its coefficient β). The strategies listed above were evaluated on the benchmark datasets MNIST, Fashion-MNIST, HAM10000 and COVID-19, using both lightweight and conventional convolutional neural networks architectures, including Custom-CNN, SBNet-CNN, ResNet18, VGG16 and DenseNet121. The empirical findings also support the idea that SMSA(x, n) is more suitable to increase convergence rates and to improve the learning of discriminative features in data-specific regimes, whereas SMSA(x, β) its adaptive counterpart is more suitable to provide better optimization stability, faster convergence, and better predictive performance. Indeed, using layer-wise SMSA in the SBNet- CNN model on the Fashion-MNIST data, the test accuracy of 97.52% was reached, which is, in fact, better than what ResNet18(94.18%) and VGG16(94.13%) had done. In addition, the layer-wise adaptation provided more robustness with imbalanced classes by achieving a classification test accuracy of 98.59% in HAM10000 and having a 4% positive change in the accuracy of COVID-19 classification with DenseNet121. These findings support the importance of the adaptive activation mechanisms which have a critical influence on the regulation of gradient dynamics, the development of nonlinear representations, and the process of generalization. Future studies will focus on generalizing SMSA to graph-based neural networks, transformer models, and self-supervised paradigms, which is complemented by a comprehensive theoretical analysis of gradient flow and representation geometry, which will eventually bring the state of the next-generation neural network design.

## Introduction

Deep learning was a revolutionary factor in enhancing artificial intelligence in the application of artificial intelligence in healthcare, medical imaging, and automated diagnostic systems. It allowed end to end learning of complex visual data, which greatly contributed to accuracy, flexibility, and clinical decision support. However, challenges such as vanishing or exploding gradients, unstable convergence, and weak generalization continued to limit the performance of deep neural networks, especially on sensitive and heterogeneous datasets [1]. With continuous, bounded, and differentiable activation functions, deep learning neural networks depict exceptional capability to approximate complex and nonlinear function between image data and true image categories. Based on the Universal Approximation Theorem, deep learning architectures can achieve arbitrary levels of accuracy when given a depth and properly organized activation dynamics. Such a theoretical background lies behind their exceptional results in a broad spectrum of visual recognition tasks, both generic performance evaluation metrics and more critical medically important visual imaging tasks [2]. Activation functions are significant in determining the complex non-linearity aproximation function and learning dynamics of neural networks, which promotes the constant development and optimization of activation learning strategies, particularly those that extend adaptability and expressivity [3]. Deep learning models are nowadays being used in high-rank image recognition and sensitive classification issues in which the relationships between inputs and outputs are very nonlinear and hard to approximate [4]. Traditional activation is prone to saturation which causes vanishing gradient making deep training of the network difficult. Functions that are not sharp like Softsign and Arctangent functions find it difficult to reproduce complex or high-frequency features. Activations are very flat such as ArcSinh and Gudermann, which offer poor sensitivity about the origin, leading to slow convergence. Unstable updates can be caused by too sharp transitions in highly steep functions such as a Logistic (with large beta). These constraints make learning less effective and limits the scope of the model to generalizing to a variety of data [5]. In order to overcome this difficulty, it is necessary to come up with adaptive activation functions having trainable parameters. These functions are supposed to provide dynamic control of important properties such as nonlinearity, limitedness, saturation and gradient action. By instantiating these adaptive mechanisms in the learning process using parameter optimization, neural networks can produce more precise, data-sensitive approximations and be more effective at problems with different and complicated data distributions.

The paper has engaged in a detailed research on the development, testing and empirical evaluation of a family of activation function named as **Symmetric Modifiable Slope Activation (SMSA)**. The three distinct configurations of these functions have been presented including manually tunable, globally adaptive, and layer wise adaptive and the purpose is to make convolutional neural networks expressive and increase their learning capacity. First fixed slope form of SMSA has allowed an explicit control over the nonlinear response by means of a static parameter and has also shown dataset specific optimal behavior. This shape has made converging it by tuning activation sensitivity based on the statistical properties of the input distribution stable. In further enhancement of the learning dynamics, an adaptive S shaped version of SMSA has been formed by incorporation of a learnable parameter, which is the β, that has been self-regulating the slope of activation and curvature in the course of training. This parameterization has maintained gradient flow across layers, alleviated vanishing gradient problems, and added smooth convexity that is consistent with the hierarchical nature of feature abstraction of deep architectures. Besides the theoretical design, a structurally efficient CNN has been created named SBNet that has been built with a moderate budget of parameters and has been tested in the Fashion MNIST data set to confirm the effectiveness of SMSA when limitations are held on model complexity. Additionally, the adaptive SMSA functional has been implemented on various high similarity and texture rich datasets such as MNIST, Fashion MNIST, HAM10000 and the COVID19 Radiography Dataset. These datasets present significant challenges of class separability because of slight interclass fluctuation. There have been experimental results that SMSA has eased the establishment of stronger decision boundaries that have allowed deep networks to achieve greater discriminative power and enhance generalization. There has been extensive cross validation and the results of classification have been compared systematically with that achieved by the use of the standard activation functions including ReLU, ELU, Swish, Mish and Leaky ReLU in various architectures. The adaptive variants of SMSA have always demonstrated better convergence behaviour, better stability in the optimisation process and accuracy on unseen data. These findings support the possible power of SMSA as an effective, data receptive activation scheme that can be used with deep learning models that process complex visual data.

## Literature review

Convolutional neural networks (CNNs) built on deep learning technologies have profoundly influenced the evolution of intelligent systems, especially in the spheres of healthcare, autonomous technologies, e-commerce, and medical diagnostics as well as in a vast number of industrial and commercial applications. In these areas, CNNs have helped in automated feature extraction, image-based diagnostics, real-time decision-making, and large-scale pattern recognition with minimal human intervention [6,7]. Although such successes have been achieved, it is frequently the case that large-scale annotated datasets are inadequate in practical applications. Most practical cases only have access to relatively small datasets, which is restricted to issues of privacy, annotation can be expensive, and domain-specific factors are limiting to size [8,9]. As a result, CNN designs in these applications are now being geared towards the ability to be small, data efficient, and can generalize well and show high accuracy even when limited data is available. The dimensions and magnitude of the dataset have played a significant role in determining the structural design of CNNs especially, its depth and number of parameters [10,11]. Traditionally, deeper CNN models have been used when there are large datasets, and high resolution and hierarchies of features may require more than just deep layers to support effective representation learning [12]. On the other hand, small or moderate-sized datasets may also have the best performance with lightweight CNN models that have fewer layers and fewer parameters that minimize overfitting and decrease computation requirements [13]. This correlation between model size and dataset size has been repeatedly confirmed by recent empirical research. The convolutional operations, implemented in deep learning systems, have resulted in the development of deep CNNs, now becoming key in image recognition tasks in a variety of application areas. These architectures are specifically crafted to grow with data complexity and resolution, so that they are especially useful in high-stakes applications like healthcare and autonomous systems. CNN models have been improved over time as they concentrate on depth optimization, parameter efficiency, and inference speed to satisfy the requirements of modern applications in terms of performance and deployment. Empirical evidence has also pointed out that parameter size has a strong effect on the predictive power of deep CNNs. Indicatively, Kainat and Mujeeb [14] tested ResNet50 on CIFAR-10 and Fashion-MNIST, where they reported higher accuracy of 86% on Fashion-MNIST compared to 73% on CIFAR-10 despite having an imbalanced data. Their findings highlighted the significance of balancing of datasets, regularization of datasets and hyperparameter optimization of datasets with the intention of improving generalization and minimizing computational expenses. Aradea *et al.* [15] proposed AdaptiveSpinalNet, a hybrid CNN, which combined adaptive kernels with SpinalNet, whereby their VGG-5 + AdaptiveSpinalNet model had 95.21 percent validation accuracy with just 1.1M parameters on Fashion-MNIST, a smaller dataset. Equally, Raza *et al.* [16] introduced a low-complexity O-CNN with NGNDG-AF activation function, which achieved 99% training and 98% validation accuracy on HAM10000, as compared to refined models like ResNet152V2. They also incorporated explainable AI techniques such as Grad-CAM and Grad-CAM++ in their model to increase its interpretability, thereby increasing its likelihood of clinical adoption. Al Mahmud *et al.* [17] optimized CNN with augmentation methods on HAM10000 and obtained 97.78% diagnostic accuracy and 97.9% precision, recall, and F2-score. Simultaneously, Hussain and Shouno [18] successfully designed the MAGRes-UNet, a framework that adds Multi-Attention Gates (MAGs) and residual links to extract fined features in medical image segmentation, the MAGRes-UNet architecture, and provided its results in medical image segmentation and regression. Their model, which was trained on CE-MRI datasets and HAM10000 datasets using Mish and ReLU activations using Adam and AdamW optimizers, significantly outperformed U-Net and ResUNet. In particular, it has reached 99.94% accuracy, 98.29% iOU, and 97.75% Dice on CE-MRI and 99.71% accuracy, 97.83% iOU, and 97.36% Dice on HAM10000. All of these findings point towards one thing, which is that CNN performance and efficiency are highly dictated by compatibility among the architectural depth, parameter size, and dataset complexity. The essence of these developments is the connection between nonlinear activities and linear changes. Although the structure of neural networks is built on affine operations, the nonlinear activation functions allow the neural network to learn and model complex hidden patterns in the data. The Universal Approximation Theorem is a proposition that the neural network with the correct number of neurons and activation functions can be utilized to approximate any continuous function within any desired accuracy. This makes the role of activation functions not only the helper functions but also central enablements of expressiveness, flexibility, and representational strength of modern CNN architectures [19]. Activation functions have a far reaching effect on various parameters of neural network dynamics, such as gradient flow, the smoothness of optimization, convergence, and the stability. They prove to be especially crucial in the context of sensitive fields like medical image analysis, where the pattern is usually faint, and datasets are extremely diverse. In this regard, the choice of activation functions is also significant in determining model performance. Functions that offer gradients that are smooth, functions with lower bounds or functions with adaptive characteristics are particularly effective in enhancing the stability of the training and the consistency of the outcomes when using generalization [20]. The paper by Ullah *et al.* [21] methodically investigated various adaptive activation functions on the Fashion-MNIST data set with VGG16. Their experiments found Tanhsoft-3 and AHerfReLU to be the most accurate with a validation accuracy of 93.91% and 93.92%, respectively. The Smish (93.53%), TanhLU (93.41%), Pserf (93.29%), and Serf (93.16%) architectures were also competitive, which demonstrated the helpfulness of tunable activations to enhance the generalization capacity of CNNs. More developments to this problem were introduced by Pandey and Srivastava [22] who introduced the Rectified Tangent Activation (RTA). RTA has performed better than traditional activations (like ReLU, Swish, ELU and Tanh) across datasets like CIFAR-100, Fashion-MNIST and Chest X-ray. It provided better accuracy, recall, F1-score and validation rates in ResNet18-based evaluation, getting 91.76% on CIFAR-100 and 78.84% on Chest X-ray. Such outcomes also underscored the flexibility, effective convergence and categorizational power of RTA. Similar results were reported by Nanni *et al.* [23], who showed that it is possible to significantly improve the biomedical image classification by changing the range of activation functions in CNN backbones. Substituting ReLU with other frameworks like SReLU, APLU, MeLU, GaLU, Mish, Swish and PDELU in ResNet50 resulted in significant performance improvements on 13 medical datasets. The wMeLU(255) model was the best stand-alone with the highest average accuracy of 85.27% compared to the standard ReLU baseline (84.55%). In addition, further improvements were achieved with ensemble approaches which relied on stochastic activation selection where StoFullAS15(255) attained the highest average accuracy of 90.31%. These results substantiate the hypothesis that diverse activations (layered) ensembles always better perform compared to fixed-activation counterparts in biomedical imaging tasks. Further contributions came out by Biswas *et al.* [24], who suggested the activation functions of SMU and SMU-1. The designs are admimations of ReLU, Leaky ReLU, Maxout and GELU with increased smoothness and flexibility. Replacement of ReLU with SMU showed significant improvements on CIFAR-100 Top-1 accuracy, improving by 6.22%, 3.39%, 3.51%, and 3.08% when used with ShuffleNet V2, PreActResNet-50, ResNet-50, and SeNet-50, respectively. This direction was developed further by the works of Harmon and Klabjan [25]. They described an approach based on an a**ctivation ensemble**. Their approach allows several activation functions to co-exist in every layer with weight parameters of interest being dynamically optimized to select the most effective activation per neuron. This multi-activation approach improved the performance of a variety of architectures and datasets, such as 99.34% to 99.40% improvement of MNIST CNNs and 95.16% to 96.28% improvement of ISOLET FFNs. These advances confirmed the strength of activation ensembles as an effective tool of enhancing plasticity and learning ability in neural networks. Jagtap *et al.* [26] proposed both neuron-wise and layer-wise adaptive activations to enhance the convergence and performance of deep and physics-informed neural networks, in which a scaling-factor is a learnable parameter of each activation, which the network can optimize by training. Their approach with an extension by a slope-recovery term in the loss function does not contain sub-optimal critical points, and their gradient flow conditions implicitly, without any explicit matrix work, thus providing more stable and faster optimization. The superiority of the Mish and AdamW configuration was statistically established by a T-test with the *p*-values being significantly less than 0.05 in all significant metrics. These findings indicate that the combination of attention mechanisms and adaptive optimization can greatly improve CNN on complex medical image classification tasks [27,28]. A significant weakness, however, still exists: most activation functions are either convergence efficient, or classification accurate, but do not typically both at the same time across a variety of architectures and datasets. This limits scalability and compromises reliability in high stakes areas like clinical diagnostics. In general, it shows that combination of transformation mathematically based, expressive activation functions, and hierarchical learning features enable CNNs to categorize highly complex image datasets with good accuracy. Concurrently, the combination of dense and rich layers can enhance further performance, but the process of determining the best configuration may be complex, and one may need to perform and optimize a large number of experiments. Such a process not only makes computations more expensive but also makes the development of models more time-consuming in the real world. Going forward, it is presumed that future developments will depend on the synergetic interaction of mathematical models, architectural research, and adaptive nonlinearities. These advancements are required to attain resource efficient, high-accuracy, and clinically accurate deep learning solutions.

## Materials and methods

The Symmetric Modifiable Slope Activation (SMSA) is a curvature-based, nonlinear activation-function specially made to meet the intractable problems of gradient attenuation and limited adjustability which plague traditional activation functions. Two complementary instantiations of SMSA have been described; each is parameterised by an integer n, which controls the slope, and an adaptive variant, which adds to the process a hyper-parameter b, which controls curvature, boundedness and gradient sensitivity. The activation has been carefully mathematically tested in order to rule out the possibility of vanishing or explosive gradients. Empirical studies indicate that it is differentiable and smooth, and thus allows the stable propagation of inputs with a wide range of magnitudes. It is the first derivative of which is continuous, and the criteria of smoothness of higher order are met, so that gradient-based optimisation algorithms can exploit local error surfaces in a way which does not have to face singularities or discontinuities. A layer-wise adaptive approach has been used to improve the flexibility of the representation. The convolutional layers choose their SMSA profile autonomously in an optimal manner, which generates a personalised dynamical behaviour through the feature hierarchies. The combination of these mechanisms, which is synergistic, results in a much expressive model. Experimental pipeline was run on GPUs-accelerated hardware, hence making it easy to train large models, converge fast and be computationally tractable. First, the mechanism was tested on a Smart Block Network (SBNet) applied to the Fashion-MNIST dataset and allowed analyzing the convergence properties and generalisation.The SMSA has been used in future works to apply to classical deep learning backbones, including VGG16, ResNet18 and DenseNet121, both in fixed and adaptive configurations. All models have been trained in the same framework of the experiment, to enable an equal comparison, where the metrics of performance have been measured based on classification accuracy, the dynamics of loss convergence, as well as the generalisation gap, thus, giving information on the strength and stability of the obtained architectures.

### Mathematical formulation of S shape activation function

The Symmetric Modifiable Slope Activation (SMSA) operation has been developed in two different versions a manually controlled one, called as SMSA(x,n), and an adaptive version called as SMSA(x,β). They are both characterized by symmetric, piecewise-smooth expressions that are meant to ensure that differentiability and gradient propagation are stable. The learnable parameter, which is illustrated as a slope and curvature modulation, can be dynamically modulated during the training process. Such fundamental properties as boundedness, adaptability, and gradient preservation have been well-developed and demonstrated in practice. According to the definition given in Eq (1), Symmetric Modifiable Slope Activation (SMSA(*x*, *n*)) is the solution to the shortcomings of the traditional activation functions, including Sigmoid, Tanh, Softsign, Arctangent, ArcSinh, Gudermann, Algebraic Sigmoid, and Logistic, as it maintains a symmetric shape around the point of *x* = 0, with an explicit slope control via the fact that the slope shape is regulated by a single parameter, which is often denoted *n*. This provides detailed control of gradients hence improving optimization dynamics in training.


$$\begin{array}{c} f_{SMSA}\left(x\right)=SMSA(x,n)=\frac{{\left(\frac{x}{n}\right)}^{2}}{1+{\left(\frac{x}{n}\right)}^{2}}\;\text{sign}(x). \end{array}$$ (1)

The variable *x* represents the input value, while *n* > 0 is a scaling parameter that controls the slope and symmetry of the function in Eq (1). Secondly the proposed **Adaptive-SMSA**(x,β) function Eq (2) is defined as


$$\begin{array}{c} F_{\text{SMSA}}(x)=SMSA(x,\beta )=\frac{x^{2}}{1+\beta x^{2}}\cdot \text{sign}(x), \end{array}$$ (2)

where β>0 is a tunable parameter and sign(*x*) is the signum function defined by


$$\begin{array}{c} \text{sign}(x)=\left\{ \begin{array}{cc} -1 & \text{if }x<0,\\ 0 & \text{if }x=0,\\ 1 & \text{if }x>0. \end{array}\right. \end{array}$$ (3)

**Fig 1:**
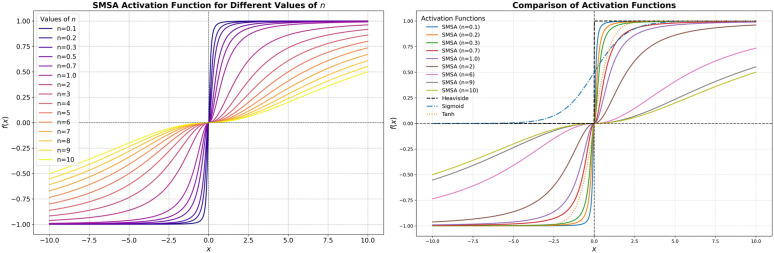
Baseline S-shaped curves of the SMSA (*x*,*n*) activation function for different values of the scaling parameter *n.*

**Fig 2:**
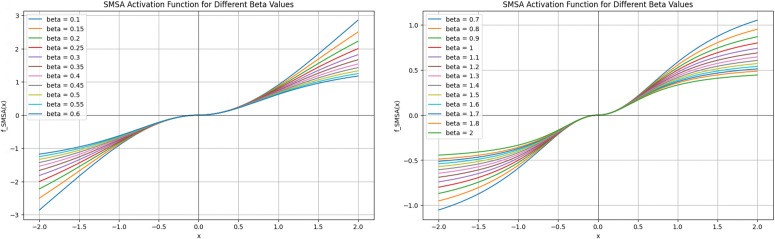
Curvature behavior of the adaptive SMSA (x,β) activation function for small values of the parameter β.

Fig 1 below demonstrates clearly that the parameter *n* in the proposed activation function entails a very specific variational control of the slope to the proposed activation function which is SMSA(x,n). The zero-gradient domain is concurrently formed and the area of the exponential exploration of the nonlinear response is carved by modulating *n*. Similarly, Figs 1 and 2 illustrate the control of concavity, range, and total non linearity by the learnable parameter of the adaptive form of SMSA, namely, SMSA(x,β). The process of fine-tuning of the value of beta therefore makes the model more flexible to diverse β network complexities and network depths.


$$\begin{array}{c} \lim_{x\to +\infty }f_{SMSA}(x)=1\;\text{and}\;\lim_{x\to -\infty }f_{SMSA}(x)=-1. \end{array}$$ (4)

$$\begin{array}{c} \lim_{x\to \pm \infty }F_{\text{SMSA}}(x)=\pm \frac{1}{\beta }. \end{array}$$ (5)

The SMSA function SMSA(x,n) exhibits **fixed boundedness** in Eq (4), saturating at ±1 regardless of the parameter *n*, as verified in Fig 1. In contrast, the adaptive form SMSA(x,β) demonstrates **dynamic boundedness** in Eq (5), saturating at $\pm \frac{1}{\beta }$, where β is a learnable parameter. This dependency on β enables flexible control over the activation range, allowing the network to adjust its nonlinearity during training, as verified in Figs 1 and 2. The analytical formulation of the derivative of the SMSA(x,n) function is given by:


$$\begin{array}{c} {f^{\prime }}_{SMSA}\left(x\right)=\frac{2\left(\frac{x}{n}\right)}{n{\left(1+{\left(\frac{x}{n}\right)}^{2}\right)}^{2}}\text{sign}(x). \end{array}$$ (6)

The first derivative $F_{\text{SMSA}}^{\prime }(x)$ has been computed for x≠0. The identity (*x*^2^) sign(*x*) = |*x*|*x* holds for all nonzero *x* and has been utilized to simplify the expression.


$$\begin{array}{c} F_{\text{SMSA}}(x)=\frac{|x|x}{1+\beta x^{2}}, \end{array}$$ (7)

then, the first derivative is for *x* > 0, |*x*| = *x*, so:


$$\begin{array}{c} F_{\text{SMSA}}^{\prime }(x)=\frac{2x(1+\beta x^{2})-2\beta x^{3}}{(1+\beta x^{2}{)}^{2}}=\frac{2x}{(1+\beta x^{2}{)}^{2}}, \end{array}$$ (8)

for *x* < 0, |x|=−x, so the result is


$$\begin{array}{c} F_{\text{SMSA}}^{\prime }(x)=\frac{-2x(1+\beta x^{2})+2\beta x^{3}}{(1+\beta x^{2}{)}^{2}}=\frac{-2x}{(1+\beta x^{2}{)}^{2}}. \end{array}$$ (9)

The derivative of SMSA(x,n) versus the independent variable x, as shown in the Eq (6) reveals that the slope intensities are controlled by a single parameter namely the parameter *n*. This kind of dependency offers explicit control of the gradient flow of the network. Adaptive formulation SMSA(x,β) has its derivatives described in Eqs (8) and (9). These derivatives are a dynamically varying entity, which can be dynamically varied by changing the parameter β during training, and can therefore enable the network to be per-layer modulators of the gradient. Convergence and optimisation of this type of modulation are possible. Curvature of SMSA is convex at the position where *x* = 0 and this facilitates a very favourable and effective gradient propagation at the early stages of training. As the magnitude of the input increases, the SMSA asymptotically switches to a concave regime thereby dispensing the risk of gradient explosions or saturation effects. It implies that the adaptive curvature process renders deep convolutional neural networks more robust because they have good learning dynamics and do not encounter the common phenomena of vanishing or exploding gradients in deep architecture.


$$\begin{array}{c} f_{SMSA}^{\prime \prime }(x)=\frac{2\left(1-3{\left(\frac{x}{n}\right)}^{2}\right)}{n^{2}{\left(1+{\left(\frac{x}{n}\right)}^{2}\right)}^{3}}\cdot \text{sign}(x). \end{array}$$ (10)

On the inputs that meet the condition $|x|<\frac{n}{\sqrt{3}}$, the second-order derivative $f_{\text{SMSA}}^{\prime \prime }(x)$ is positive, which proves that the function is located in a convex regime. On the other hand, when the value of x exceeds the $|x|>\frac{n}{\sqrt{3}}$ of three, the second derivative becomes negative, meaning that the shape is concave. Such a concavity/ convexity transfer gives SMSA the capability to strongly adapt the sensitivity of the gradient, and hence to promote stable optimisation in deep network designs.


$$\begin{array}{c} F_{\text{SMSA}}^{\prime \prime }(x)=\frac{4(1+\beta x^{2})(1+\beta x^{2}-2\beta x^{2})}{(1+\beta x^{2}{)}^{4}}\cdot \text{sign}(x)=\frac{4(1-\beta x^{2})}{(1+\beta x^{2}{)}^{3}}\cdot \text{sign}(x). \end{array}$$ (11)

The second derivative of the adaptive SMSA has the second term in the Eq (11), which has been used to obtain its curvature as shown in the equation below: In case the input is such that it satisfies: $|x|<\frac{1}{\sqrt{\beta }}$ the numerator: $\left(1-\beta x^{2}\right)$ is strictly positive and hence the second derivative is $F_{\text{SMSA}}^{\prime \prime }(x)>0$, and thus the deriving is conclusive as to a convex behaviour. Conversely, at a value of $|x|>\frac{1}{\sqrt{\beta }}$, the value of $\left(1-\beta x^{2}\right)$ is negative, and $F_{\text{SMSA}}^{\prime \prime }(x)<0$, thus exhibiting concave behaviour. The inflection point, at $|x|=\frac{1}{\sqrt{\beta }}$, where convexity changes to concavity, as $F_{\text{SMSA}}^{\prime \prime }\left(\pm \frac{1}{\sqrt{\beta }}\right)=0$. This learnable parameter controlled adaptive curvature modulation allows the SMSA to adapt flexibly to nonlinear patterns by dynamically changing the convexity and concavity in the course of the optimisation process.

The proposed combineds SMSA(x,n) function, as illustrated in Table 1 and Fig 1, generalizes classical S-shaped activation functions by skillfully implementing at least Sigmoid, Tanh, Softsign, and similar functions by a single tunable parameter, *n*. This flexibility allows the nonlinearity to be under control dynamically thus making the gradient flow rapid and stable convergence in training. This in turn increases the learning capacity and optimisation of deep neural networks of the SMSA.

**Table 1:** Comparison of SMSA with classical S-shaped activation functions.

Function	SMSA *n*	Range	Symmetry	Sharpness
Sigmoid	≈0.5	(0, 1)	No	Medium
Tanh	≈0.6	(−1,1)	Yes	High
Softsign	1−2	(−1,1)	Yes	Medium
Arctangent (scaled)	1−2	(−1,1)	Yes	Low
ArcSinh (normalized)	2−3	(−1,1)	Yes	Very Low
Gudermann (scaled)	0.7−1	(−1,1)	Yes	Low
Algebraic Sigmoid	0.5−0.7	(−1,1)	Yes	Low
Logistic (β=2)	≈0.3	(0, 1)	No	Very High

### Layer-wise adaptive learning strategy

In modern deep learning studies, it has been established that neural networks have architectures to discover hierarchical encodings of features, where early layers learn simple patterns, and later layers learn more abstract semantic features [29,30]. Both the nonlinear transformation of each constituent depth and, as a result, the inherent properties of the activation function can have a controlling effect on the abstraction properties of the network, and the geometry of representations at that depth. It is on this basis that in order to realize the representational potential of each stratified layer to a full extent, nonlinearities specifically designed to display curvature, smoothness, and flexibility characteristics relevant to the needs of the functional requirements of the layer in question are essential. The adaptive parameter of each convolutional layer is a convolutional layer-specific parameter, namely, the parameter, denoted by $\beta_{i}^{\left(l\right)}$, thus permitting the layer-specific non-linear modulation process that refines the abstraction of the features gradually, starting with fine-grained local patterns, and progressing to more complex and high-level semantic features. This type of dynamic adjustment has been shown to enhance classification accuracy in problems which are sensitive to deep, hierarchical representation learning, such as object recognition, structured image analysis, etc.


$$\begin{array}{c} b_{k,i}^{\left(l\right)}=\Phi_{i}^{\left(l\right)}(x_{k};\;\beta_{i}^{\left(l\right)}) \end{array}$$ (12)

The network has a specific, learnable parameter assigned to each layer, namely, the *l* layer of the network, denoted as, in a given mini-batch training epoch, the *i*^th^ pass through the network, the learnable parameter is denoted as $\beta_{i}^{\left(l\right)}$. It is a parameter that governs the non-linear dynamics of the activation function, ‘$\Phi_{i}^{\left(l\right)}$ in the layer. The value of each of the activation parameters is modified to a new one, i.e., $\beta_{j}^{\left(l\right)}$, as the process progresses to the next epoch, $\Phi_{j}^{\left(l\right)}$. Therefore, the nonlinearity injected by the functional, namely denoted as $\Phi_{j}^{\left(l\right)}$ changes over training epochs hence facilitating the network to re-adjust its nonlinear transformation properties in a direction that reflects the current direction of the learning activity. The Stochastic Mapping Search Algorithm in Eq (2) with Eq (12) as a whole is a probabilistic meta-heuristic optimisation framework. It searches a complex search space systematically by transforming stochastically and adapting mapping models, hence finding convergence to either optimal or near-optimal solutions by a prudent tradeoff of exploratory and exploitative search.

### Architectures: SBNet-CNN, Optimized CNN, ResNet18, VGG16 and DenseNet121

This article has conducted a methodical alteration of the conventional convolutional neural network (CNN) systems to determine the effectiveness of the proposed Symmetric Modifiable Slope Activation (SMSA) on various data. Specifically, a multi-block CNN [16] was used on HAM10000, ResNet18 and VGG16 on Fashion-MNIST, and DenseNet121 [31] on X-ray images of COVID-19. In both scenarios, the regular activation functions were replaced with SMSA variants, and adaptive strategies, such as the layer-wise and global schemes, were added to increase the flexibility of the training. In the case of MNIST, a lightweight CNN was trained using SMSA to achieve computational efficiency, and the SBNet -CNN architecture was used in Fashion-MNIST to incorporate layer-wise adaptive SMSA to learn scalable nonlinearity. The SBNet- CNN in Tables 2–4, to be described with a **593,665 parameter 256-filter convolutional block,** which was then followed by adaptive SMSA, pooling, and dropout layers. Later intermediate layers added further **1.77M parameters** to the initial set of feature layers by repeated 256 channel convolution, to increase feature separation. The fully connected stage was then flattened into a 2304-dimensional vector then comprised of a 64-neuron layer with 147, 520 parameters and a 128-neuron layer with 8, 320 parameters, both with SMSA, batch normalization (512 parameters) and dropout. The network ended with a 10-class Softmax output whose output layer has 1,290 parameters. Generally, SBNet -CNN repeatedly used SMSA at every layer, which combined adaptivity, nonlinearity, and regularization to produce strong performance on Fashion -MNIST.

**Table 2:** Model architecture with Input and initial convolutional layers of SBNet-CNN.

Layer (type)	Output Shape	Parameters #
input_layer_1 (InputLayer)	(None, 28, 28, 1)	0
conv2d_5 (Conv2D)	(None, 28, 28, 256)	2,560
smsa_1 (SMSAActivation)	(None, 28, 28, 256)	1
conv2d_6 (Conv2D)	(None, 28, 28, 256)	590,080
smsa_2 (SMSAActivation)	(None, 28, 28, 256)	1
batch_normalization_5 (BatchNormalization)	(None, 28, 28, 256)	1,024
max_pooling2d_3 (MaxPooling2D)	(None, 14, 14, 256)	0
dropout_5 (Dropout)	(None, 14, 14, 256)	0

**Table 3:** Model architecture: Intermediate convolutional layers of SBNet-CNN.

Layer (type)	Output Shape	Parameters #
conv2d_7 (Conv2D)	(None, 14, 14, 256)	590,080
smsa_3 (SMSAActivation)	(None, 14, 14, 256)	1
conv2d_8 (Conv2D)	(None, 14, 14, 256)	590,080
smsa_4 (SMSAActivation)	(None, 14, 14, 256)	1
batch_normalization_6 (BatchNormalization)	(None, 14, 14, 256)	1,024
max_pooling2d_4 (MaxPooling2D)	(None, 7, 7, 256)	0
dropout_6 (Dropout)	(None, 7, 7, 256)	0
conv2d_9 (Conv2D)	(None, 7, 7, 256)	590,080
smsa_5 (SMSAActivation)	(None, 7, 7, 256)	1
batch_normalization_7 (BatchNormalization)	(None, 7, 7, 256)	1,024
max_pooling2d_5 (MaxPooling2D)	(None, 3, 3, 256)	0
dropout_7 (Dropout)	(None, 3, 3, 256)	0

**Table 4:** Model architecture: Fully connected layers and output of SBNet-CNN.

Layer (type)	Output Shape	Parameters #
flatten_1 (Flatten)	(None, 2304)	0
dense_3 (Dense)	(None, 64)	147,520
smsa_6 (SMSAActivation)	(None, 64)	1
batch_normalization_8 (BatchNormalization)	(None, 64)	256
dropout_8 (Dropout)	(None, 64)	0
dense_4 (Dense)	(None, 128)	8,320
smsa_7 (SMSAActivation)	(None, 128)	1
batch_normalization_9 (BatchNormalization)	(None, 128)	512
dropout_9 (Dropout)	(None, 128)	0
dense_5 (Dense)	(None, 10)	1,290
output_cat (Activation)	(None, 10)	0

In Table 5, we have presented an architecture that uses a DenseNet121 backbone with adaptive activation of SMSA to categorize the COVID-19 radiographic images. A foundational model, which is instantiated without the use of pretrained weights, has been parametrized, and the rest, having the final twenty layers, have been parametrized with globally shared learnable coefficients of the nonlinearity (in the form of β), thus allowing the nonlinearity to be modulated across the network. This core has been followed by series of fully connected layers with each being followed by an SMSA transformation and dropout operation in order to support task-specific feature extraction. Such a composite layout allows a controlled gradient spread and fast convergence in the presence of complicated medical pictures, due to the integration of global flexibility in the deeper layers of the network.

**Table 5:** CNN architecture and custom layers in DenseNet121 with adaptive SMSA.

Layer Type	Details
Base Model	DenseNet121 (top removed, no pretrained weights)
Input Shape	224 × 224 × 3
Global Feature Extraction	Global Average Pooling
Fully Connected Layers	Dense(64) → SMSA → Dropout(0.3)
	Dense(32) → SMSA → Dropout(0.3)
	Dense(16) → SMSA → Dropout(0.3)
Activation Function	Adaptive SMSA, β∈[0.1,5] (learnable)
Output Layer	Dense(num_classes = 2), Softmax activation
Trainable Layers	First 300 layers frozen, rest trainable

**Fig 3:**
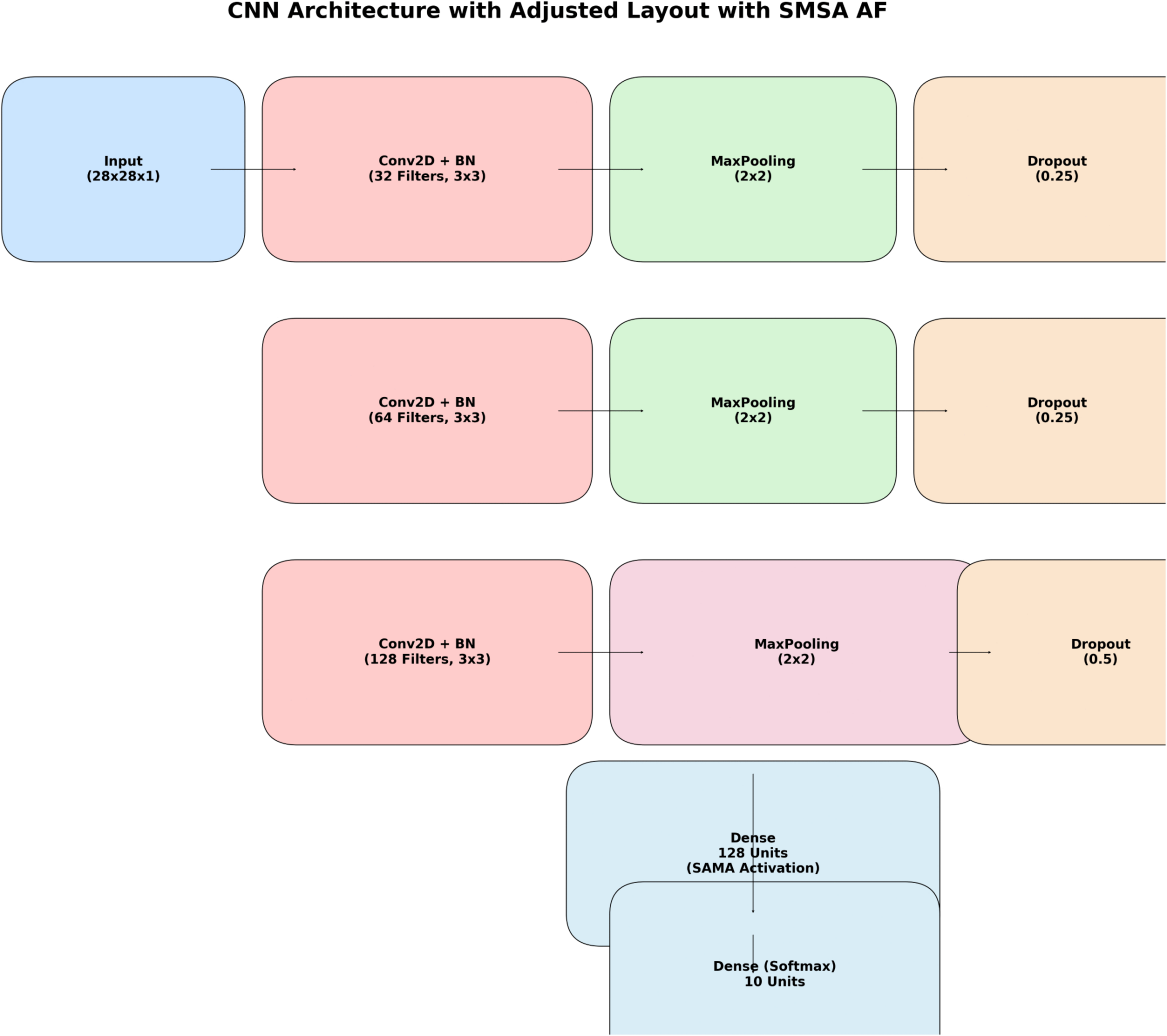
View of convolutional neural network architecture of Custom-CNN.

The CNN customized to the present study is a simplified version of Fig 3 of this work, keeping the number of layers and filter size constant. It is made of convolutional layers, each followed by SMSA(x, n), which are separated by max-pooling and finally by fully connected layers, which are task-oriented to the classification problem.

#### Mathematical formulation and design of custom CNN architecture for MNIST dataset.

The MNIST dataset consists of grayscale images with a spatial resolution of 28×28. Each image can be represented as a two-dimensional matrix:


$$\begin{array}{c} X=[\begin{array}{cccc} x_{11} & x_{12} & ... & x_{1,28}\\ x_{21} & x_{22} & ... & x_{2,28}\\ \vdots & \vdots & \ddots & \vdots \\ x_{28,1} & x_{28,2} & ... & x_{28,28} \end{array}], \end{array}$$ (13)

where $x_{ij}$ denotes the grayscale pixel intensity normalized within the range [0,1].

where Ft denotes the input feature vector, and $W^{\left(k-2\right)}$, $W^{\left(k-1\right)}$, *W*^(*k*)^ represent the trainable weight matrices at successive layers, with $b^{\left(k-2\right)}$, $b^{\left(k-1\right)}$, and *b*^(*k*)^ as their respective biases. The adaptive SMSA activation function *f*_SMSA_ is applied element-wise in each hidden layer, enabling non-linear transformations. The terminal output is derived through the use of the Softmax function that normalizes the raw logit values into a probabilistic distribution of classes, hence, making it possible to make multi-class decisions using the function with a high discriminative power. In order to describe the forward-propagation dynamics of the proposed architecture, it is convenient to write down the final output concisely as:


$$\begin{array}{c} P={\left[p_{s,k}\right]}^{1\times C}=f_{\text{Softmax}}\left(W^{\left(k\right)}f_{\text{SMSA}}\left(W^{\left(k-1\right)}f_{\text{SMSA}}\left(W^{\left(k-2\right)}Ft+b^{\left(k-2\right)}\right)+b^{\left(k-1\right)}\right)+b^{\left(k\right)}\right), \end{array}$$ (14)

### Experimental setup

This section presents the datasets, network architectures, training configuration, and computational resources used to evaluate the proposed Symmetric Modifiable Slope Activation (SMSA) and its adaptive variant. The overall aim is to perform a stringent test of behavioural properties of the model on a range of convolutional neural network architectures and test datasets, both general image classification and medical imaging specialised tasks.

#### Datasets included MNIST, Fashion-MNIST, HAM10000, Covid19.

The data represented by images that are used in computer-vision activities are by definition multi-dimensional arrays, with each dimension addressing either spatial, chromatic or intensity concepts that are necessary to visual representation. By feeding these high-dimensional image tensors to deep CNNs, the models are approximated to the respective labels by learning hierarchical feature patterns successively in each layer. However, due to the visual similarity that is common in pictures of different classes in a given dataset, classification problems may end up being more and more difficult, requiring strong feature-extracting and discrimination approaches. Four separate datasets were used in order to assess the generalizability of SMSA comprehensively:

- **MNIST**: An extremely popular benchmark containing 70, 000 grayscale images of handwritten digits (60, 000 to be used during training, 10,000 to be used during testing), each of size 28x28 pixels, and grouped into ten digits categories.
- **Fashion-MNIST**: Fashion-MNIST has a similar format, but images of fashion products (e.g., shirts, shoes, bags) of ten categories, a more challenging task, as the similarity between classes is greater.
- **HAM10000**: The Human-Against-Machine dataset with 10000 training images, which is composed of 10015 pigmented lesion dermoscopic images of 7 classes. Its medical significance and biased classes make it an attractive standard of real-world application in healthcare. Table 6 contains the information on the preprocessing pipeline that consisted of oversampling methods, normalisation measures, systematic dataset reorganisation, and sequential train-test partitioning procedures.

**Table 6:** Dataset details of HAM10000 after oversampling, normalization, and splitting.

Process Step	Description	Shape / Details
Original Data	Input images before preprocessing	Variable (imbalanced classes)
Oversampling	RandomOverSampler applied to balance class distribution	Total samples after resampling: 46,935
Reshape	Reshaped to CNN input format	(46935, 28, 28, 3)
Label Encoding	One-hot encoding using to_categorical	(46935, 7)
Normalization	Standardization: $x=\frac{x-\mu }{\sigma }$	Mean-centered with unit variance
Train-Test Split	80% training and 20% testing	Train: 37,548, Test: 9,387
Y_Train Shape	One-hot encoded labels for training data	(37548, 7)

- **COVID-19 Chest X-Ray Dataset**: Collection of X-ray images, which are thoroughly checked and include confirmed COVID-19 pneumonia, other pneumonia cases, and normal pulmonary scans. Images were resized to 224×224×3 and augmented with rotation, flipping, and brightness scaling to reduce overfitting. The preprocessing details of COVID-19 Chest X-Ray Dataset in Table 7

**Table 7:** Dataset and preprocessing details of COVID-19.

Aspect	Details
Dataset Source	COVID-19 Radiography Database (Kaggle)
Classes Used	COVID, Viral Pneumonia
Image Type	RGB Chest X-ray Images
Image Size	224 × 224 × 3
Preprocessing	Histogram Equalization (RGB), Normalization to [−1, 1]
Augmentation	Horizontal Flip, Rotation ($\pm 2^{\circ }$), Shear: 0.02, Zoom: 0.02
Dataset Split	80% Training, 20% Validation

The MNIST, Fashion-MNIST, HAM10000, and the COVID19 HR dataset collections offer a unique set of classification tasks that are challenging due to the different class compositions, the hidden similarities among classes and the complexity they possess. MNIST and Fashion-MNIST are balanced by nature, whereas HAM10000 and COVID-19 HR are highly imbalanced, which poses more challenges to both training and evaluation. These nonhomogeneous data distributions provide an effective testing environment in evaluating the flexibility and representational capability of the suggested SMSA functions. SMSA has demonstrated a remarkable capability to adjust the non-linearity control parameters, n and β, on-the-fly, which allows convolutional neural networks to be accurate approximators in a repertoire of tasks. The ability allows establishing class-specific but overlapping decision boundaries, which is critical to the modeling of the subtle differences to occur in complex datasets. It follows that SMSA has a wider range of applicability by increasing CNN generalization, even when there is high ambiguity in classification.

#### Theoretical analysis on categorical cross entropy loss, gradient flow and optimization smoothness.

The sparse categorical cross-entropy loss function is frequently used in multi-class classification tasks where target labels are represented as class indices. It measures the cross-entropy between the true label distribution *y* and the predicted probability distribution *p*, where *p* is obtained through the Softmax activation function. The vector *y* typically encodes the correct class as a one-hot vector, while *p* provides the model’s estimated probabilities for each class. This loss function has been derived from the general cross-entropy formulation and serves as a standard choice for classification problems involving mutually exclusive classes. Sparse Categorical Crossentropy Loss is defined as


$$\begin{array}{c} L=\frac{-\sum_{s=1}^{N}1\sum_{k=1}^{c}y_{s,k}\log(p_{s,k})}{N}, \end{array}$$ (15)

from Eq (14) and $p_{s,k}$ defined as


$$\begin{array}{c} \begin{array}{c} p_{s,k}=f_{Soft\max}(z_{s,k}),\\ \frac{dp_{s,k}}{d(z_{s,k})}=p_{s,k}(1-p_{s,k}). \end{array} \end{array}$$ (16)

the chain rule is again applied to compute the gradient of the loss with respect to the parameters of the last hidden layer, as shown in Eqs. (14) and (15). the weighted sum is given by $ft_{i,j}=w_{i,k}z_{i,j}+b_{j}$. Its gradient with respect to the weight $w_{i,k}$ is $z_{i,j}$, and with respect to the bias $b_{j}$, it is 1. Use results from Eqs. (1)–(16).


$$\begin{array}{c} \frac{\partial L}{\partial w_{i,j}}=\left[\begin{array}{c} \frac{(ft_{s,i})(w_{s,k})\left({\left(p_{s,k}\right)}^{2}-p_{s,k}\right)\sum_{s=1}^{N}\frac{1}{p_{s,k}}}{N}\\ \times \left\{ \begin{array}{c} \frac{2(\frac{z_{s,j}}{n})}{n{\left(1+{\left(\frac{z_{s,j}}{n}\right)}^{2}\right)}^{2}}\;\;if\;\;z_{s,j}\geq \;0,\\ \frac{-2(\frac{z_{s,j}}{n})}{n{\left(1+{\left(\frac{z_{s,j}}{n}\right)}^{2}\right)}^{2}}\;\;if\;\;z_{s,j}\;<\;0.\; \end{array}\right. \end{array}\right], \end{array}$$ (17)

$$\begin{array}{c} \frac{\partial L}{\partial b_{s,j}}=\left[\begin{array}{c} \frac{(w_{s,k})\left({\left(p_{s,k}\right)}^{2}-p_{s,k}\right)\sum_{s=1}^{N}\frac{1}{p_{s,k}}}{N}\\ \times \left\{ \begin{array}{c} \frac{2(\frac{z_{s,j}}{n})}{n{\left(1+{\left(\frac{z_{s,j}}{n}\right)}^{2}\right)}^{2}}\;\;if\;\;z_{s,j}\geq \;0,\\ \frac{-2(\frac{z_{s,j}}{n})}{n{\left(1+{\left(\frac{z_{s,j}}{n}\right)}^{2}\right)}^{2}}\;\;if\;\;z_{s,j}\;<\;0.\; \end{array}\right. \end{array}\right], \end{array}$$ (18)

$$\begin{array}{c} \frac{\partial L}{\partial w_{j,k}}=\left[\begin{array}{c} \frac{\left({\left(p_{s,k}\right)}^{2}-p_{s,k}\right)\sum_{s=1}^{N}\frac{1}{p_{s,k}}}{N}\\ \times \left\{ \begin{array}{c} \frac{{\left(\frac{z_{s,j}}{n}\right)}^{2}}{1+{\left(\frac{z_{s,j}}{n}\right)}^{2}}\;\;if\;\;z_{s,j}\geq \;0,\\ \frac{-{\left(\frac{z_{s,j}}{n}\right)}^{2}}{1+{\left(\frac{z_{s,j}}{n}\right)}^{2}}\;\;if\;\;z_{s,j}\;<\;0.\; \end{array}\right. \end{array}\right], \end{array}$$ (19)

$$\begin{array}{c} \frac{\partial L}{\partial b_{s,k}}\;\;\;\;\;=\;\;\;\frac{\left({\left(p_{s,k}\right)}^{2}-p_{s,k}\right)\sum_{s=1}^{N}p_{s,k}}{N}. \end{array}$$ (20)

#### Training details, hyperparameters, optimizers, schedulers.

Table 8 outlines the complete training protocol for mentioned CNNs model equipped with Adaptive SMSA activation on the MNist, Fashion MNist, Ham100000 and COVID-19 datasets. The training process has utilized the Adam optimizer with an initial learning rate of $1\times 10^{-4}$, which is annealed using a cosine scheduler to ensure smooth convergence. Categorical Cross-Entropy has been employed as the loss function to minimize classification error across multiple classes. To stabilize gradients and improve optimization, gradient clipping and weight decay have been integrated. Further, the SMSA activation mechanism involves an adaptively modulated β-parameter, and the time dependence of the β-parameter is recorded during training thus the time dependence gives the non-linear dynamics of adaptation in an efficient form.

**Table 8:** Comprehensive Training Setup and Evaluation Metrics for DenseNet121 with Adaptive SMSA on COVID-19 Dataset.

Training Aspect	Details
Batch Size	32
Number of Epochs	100, 50, 40
Optimizer	Adam (Learning Rate = 1e-4)
Weight Decay	$1\times 10^{-5}$
Learning Rate Scheduler	CosineAnnealingLR (Min LR = 1e-6)
Loss Function	Categorical Cross-Entropy
Activation Function	Adaptive Symmetric Modifiable Slope Activation (SMSA)
SMSA Hyperparameters	Adaptive β (Initial Value = 1.0, Range: [0.1, 5.0])
Gradient Clipping	Applied with Threshold = 1.0
Metrics Monitored	Accuracy, Precision, Recall, F1-Score
Callbacks	*EarlyStopping* (Patience = 10), β Trend Logger
Evaluation Methods	Confusion Matrix, Classification Report, ROC Curve
Plotting	Accuracy, Loss, Precision, Recall, F1-Score, β Trend Curves
Precision Mode	Full Precision (FP32)
Model Saving	Best Model Weights Restored via EarlyStopping

#### Evaluation metrics: Definitions and mathematical formulation.

The effectiveness of the suggested activation in CNN frameworks was measured using six classic measures of performance listed in Table 9. These measures are accuracy, precision, recall, F1-score, convergence rate and computation cost. Accuracy refers to the percentage of correct findings in general whereas on the other hand, precision and recall measure the ability of the model to reduce false positive and false negative findings, respectively. The F1-score provides a harmonised approach because it combines precision and recall. The convergence speed is a measure of the speed with which the model converges in training, and computational cost is a measure of the efficiency of training as indicated by the training time, memory requirements, and floating-point operations. Taken together these measures provide the overall assessment of the faithfulness of classification, dynamics of learning and the efficiency of resources.

**Table 9:** Impact of Activation Functions on CNN Evaluation Metrics.

Metric	Interpretation	Activation Function Impact
Accuracy	Overall correctness	Enhanced feature learning
Precision	Correctness of positive predictions	Reduces false positives
Recall	Ability to capture all positives	Minimizes false negatives
F1-Score	Balance of precision and recall	Uniform classification robustness
Convergence Speed	Epochs to stability	Gradient smoothness accelerates learning
Computational Cost	FLOPs, Time, Memory	Simplicity vs. Complexity trade-off

## Results and discussion

This part provided a rigorous examination of the empirical information that corresponds to the mechanism of Symmetric Modifiable Slope Activation (SMSA) and, through it, project our findings onto the current literature on the design of activation functions to deep learning. The empirical experiment, which entails an all-encompassing gathering of benchmark exercises, will likewise demonstrate a constructive variation in the performance as compared to the canonical nonlinearities such as ReLU, ELU, and Swish. This improvement is apparent in shallow convolutional designs as well as in deep residual networks therefore attesting to the flexibility of SMSA. Besides the conventional performance metrics, there is an underlying sensitivity analysis of gradient slope hyperparameter, which is directly proportional to the gradient flow, which in turn influences the speed of convergence and the generalization capability. The relationships between hyperparameter optimisation and learning dynamics have a complex relationship which is explained by this observation. Finally, the paper describes the efficiency of SMSA through a sequence of statistical perturbation experiments, which meticulously shows how it works in a wide spectrum of architectural paradigms. The overall findings confirm the value of the curvature-managed activations in the formation of representation-learning paths and in stabilisation of the optimisation in the convolutional neural-network settings.

Ablation study on gradient flow dynamics, range modulation, and nonlinearity characteristics

The graphics presented in Figs 4–17 have been summarised in Table 10 and reflect the experiment results of the (Eq. 1)’s slope control parameter n across the MNIST, Fashion-MNIST, and HAM10000 databases. The tuning of n has continuously determined convergence behavior, performance (computational efficiency), and generalization (training-validation accuracy curves that overlap) performance at all sets of data.

**Fig 4:**
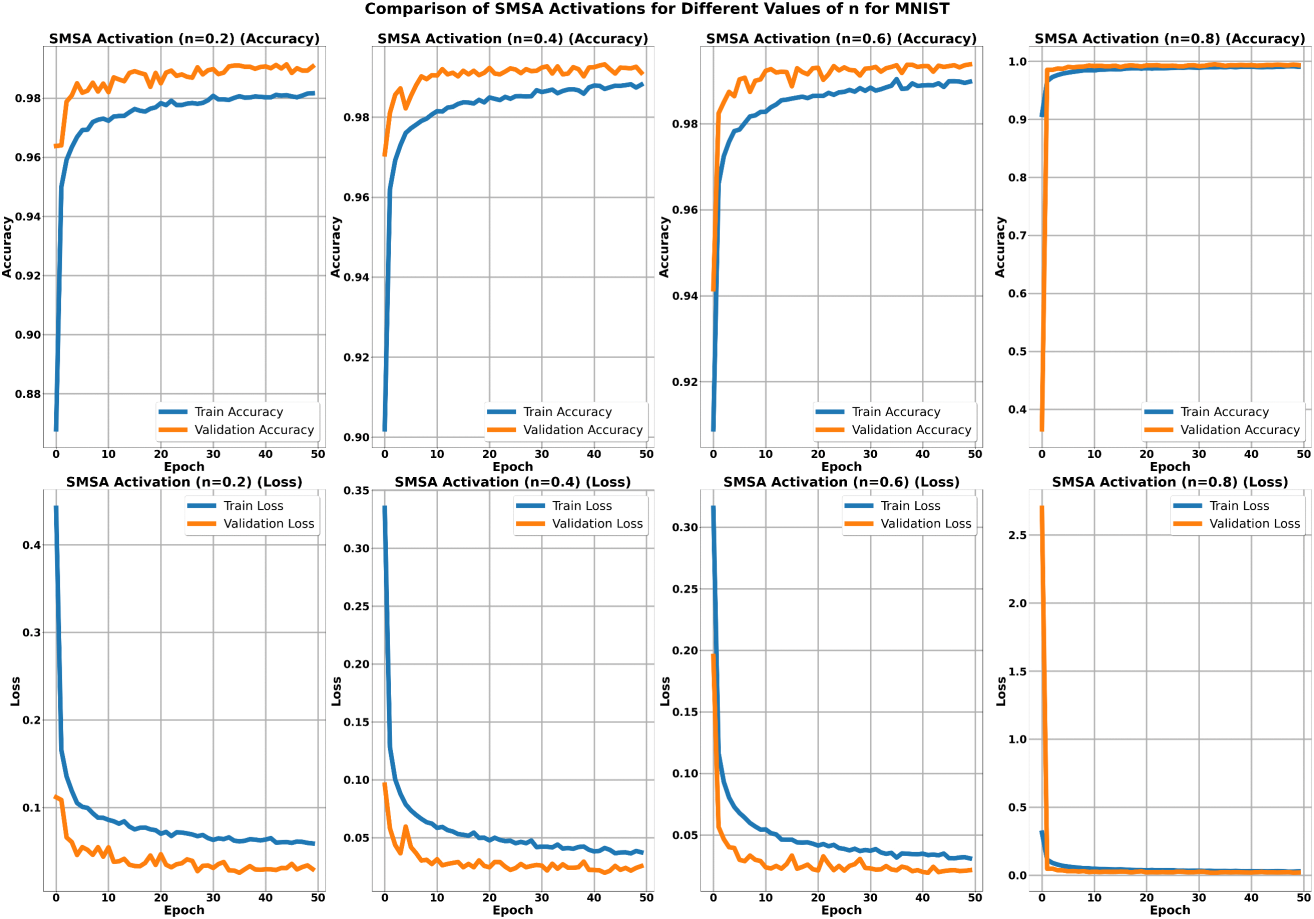
Effect of lower values of the SMSA scaling parameter (*n*) on model convergence and performance metrics. Changes in loss and accuracy trends are observed during training and validation.

**Fig 5:**
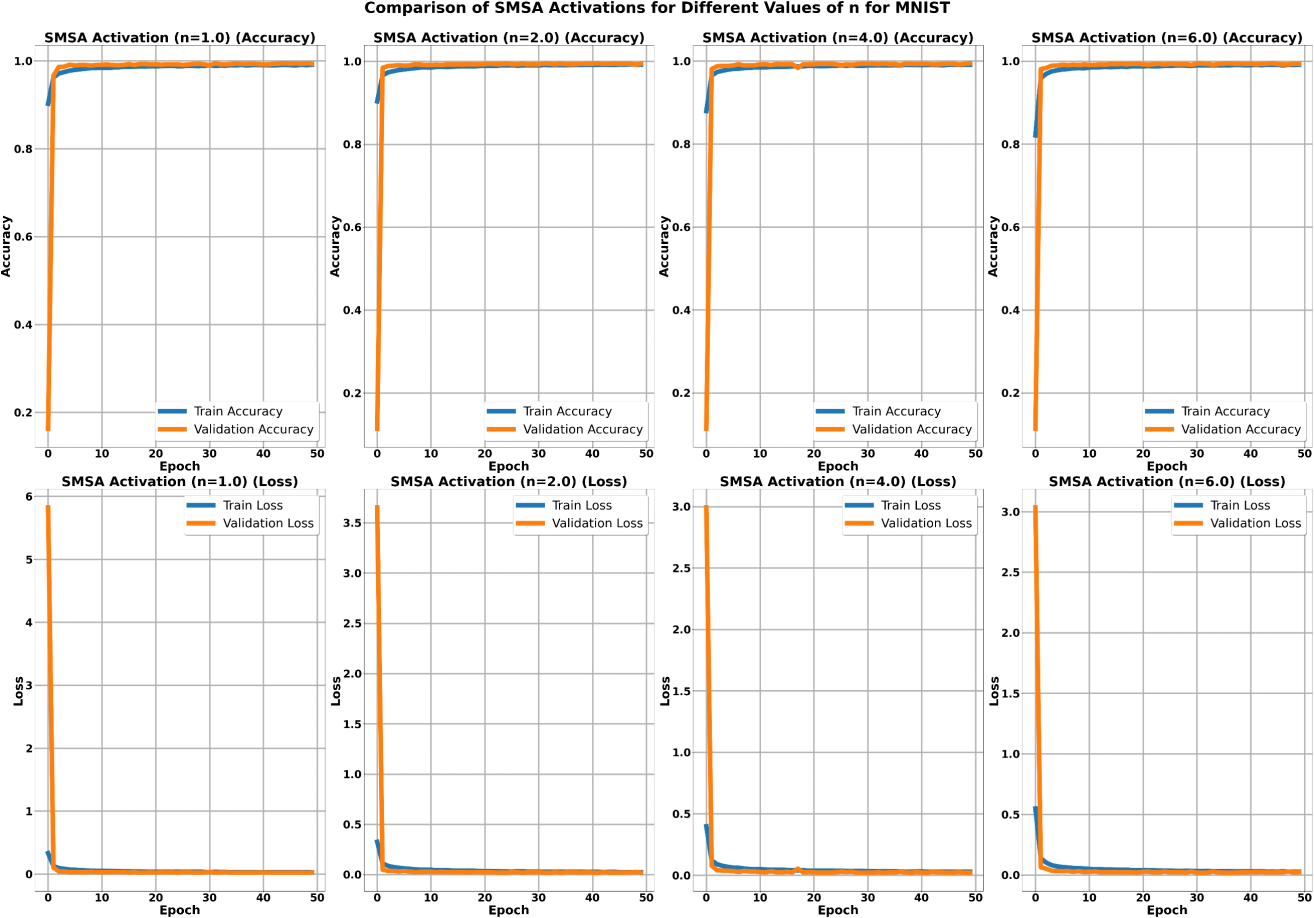
Effect of higher values of the SMSA scaling parameter (*n*) on convergence behavior and performance metrics. Variations in training and validation loss and accuracy are observed as *n* increases.

**Fig 6:**
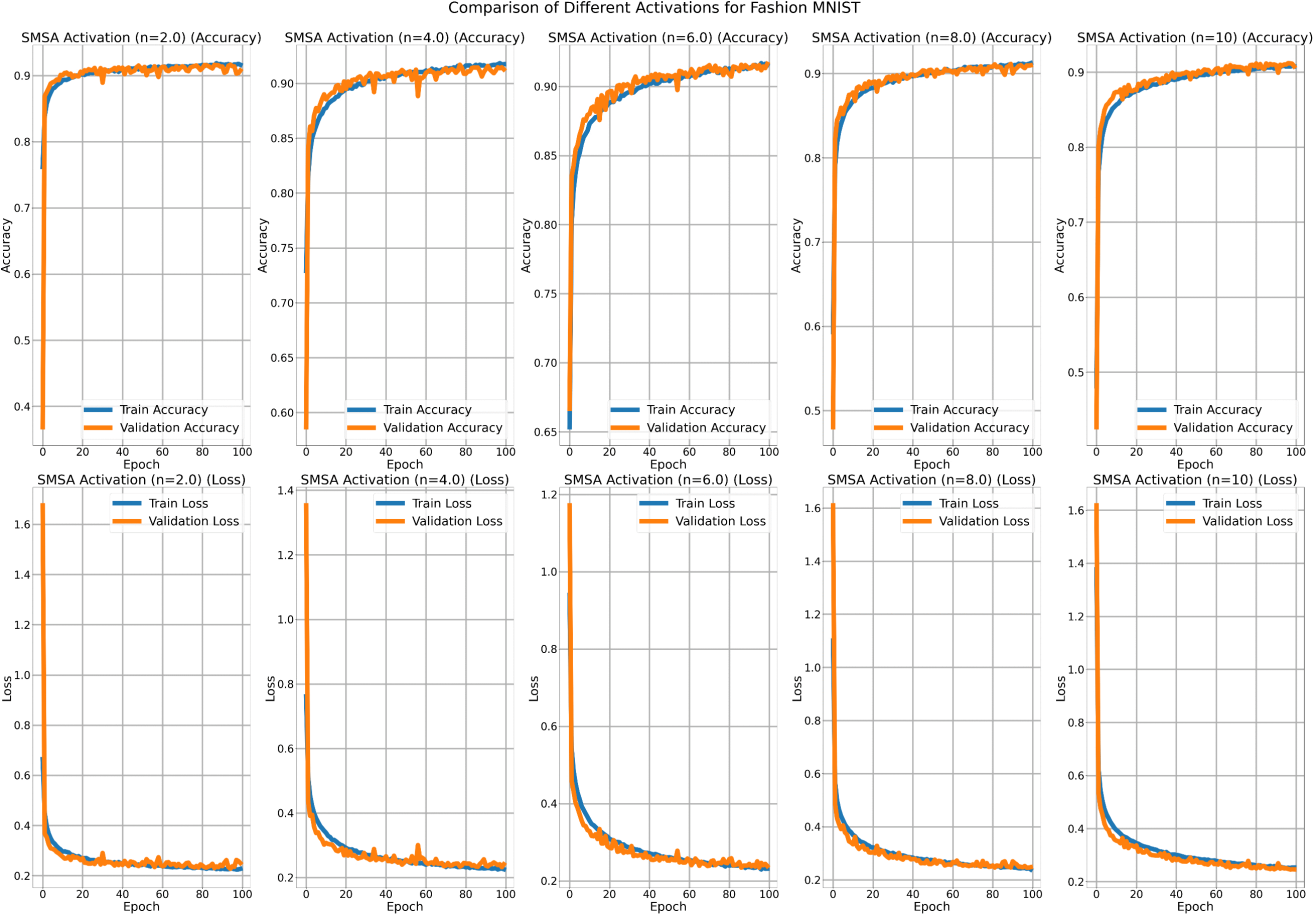
Effect of increasing values of the SMSA scaling parameter (*n*) on model performance on the Fashion-MNIST dataset. Changes in training and validation loss and accuracy are observed as *n* varies.

**Fig 7:**
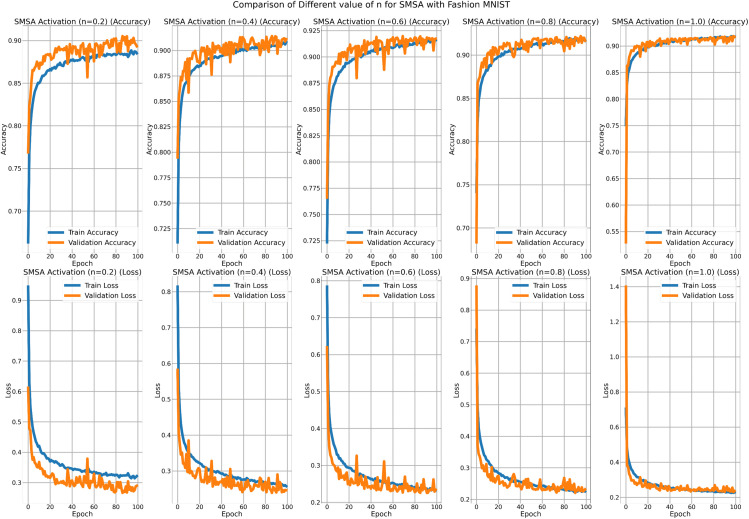
Effect of higher values of the SMSA scaling parameter (*n*) on training dynamics and performance metrics. Variations in convergence behavior, loss, and accuracy are observed as *n* increases.

**Fig 8:**
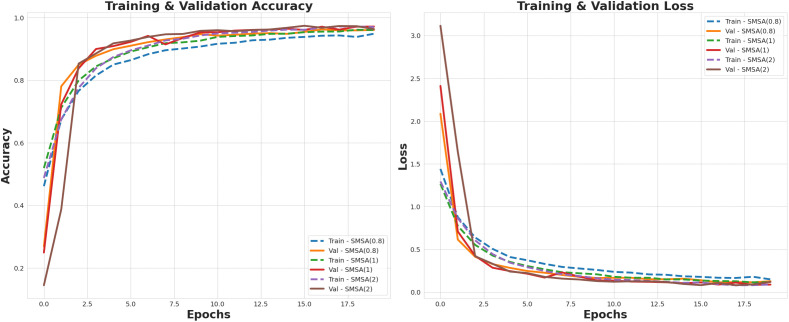
Effect of different values of the SMSA scaling parameter (*n* = 0.8, 1, 2) on gradient behavior and performance of the optimized CNN model on the HAM10000 dataset.

**Fig 9:**
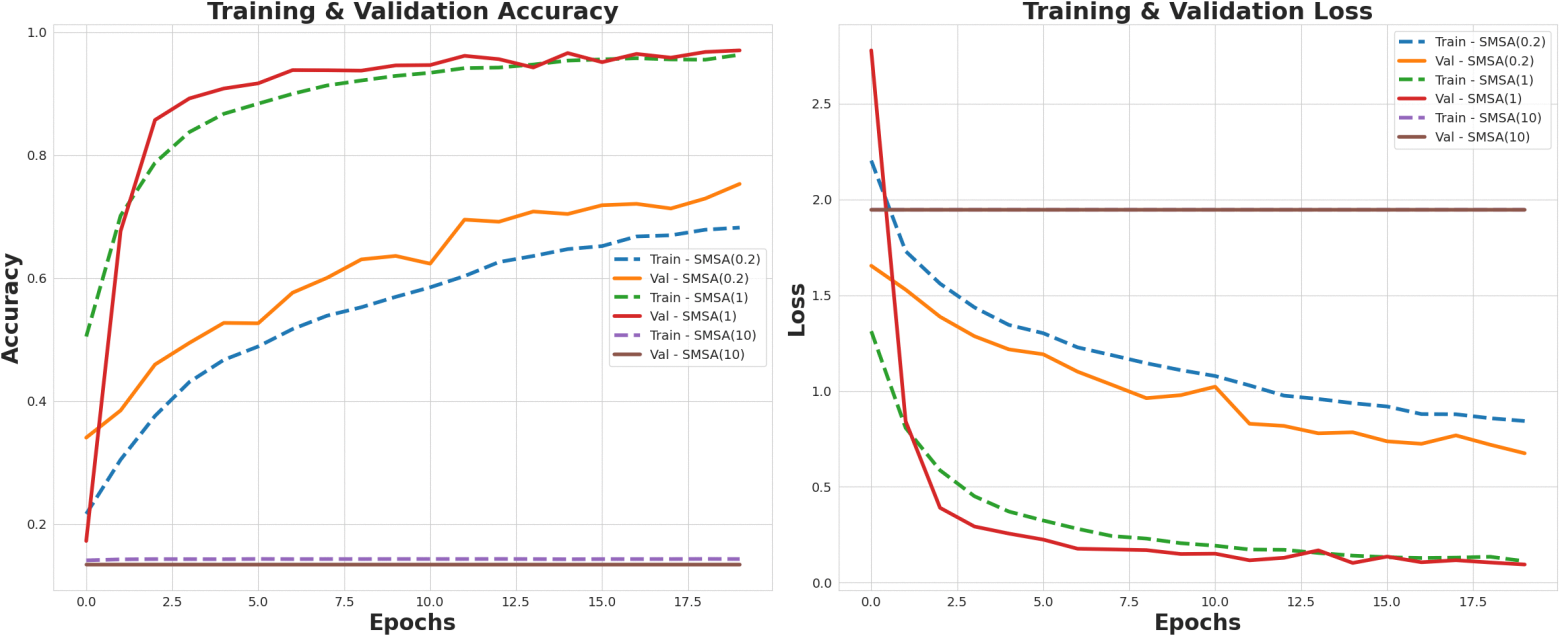
Effect of different values of the SMSA scaling parameter (*n* = 0.2, 1, 10) on gradient behavior and performance of the optimized CNN model on the HAM10000 dataset.

**Fig 10:**
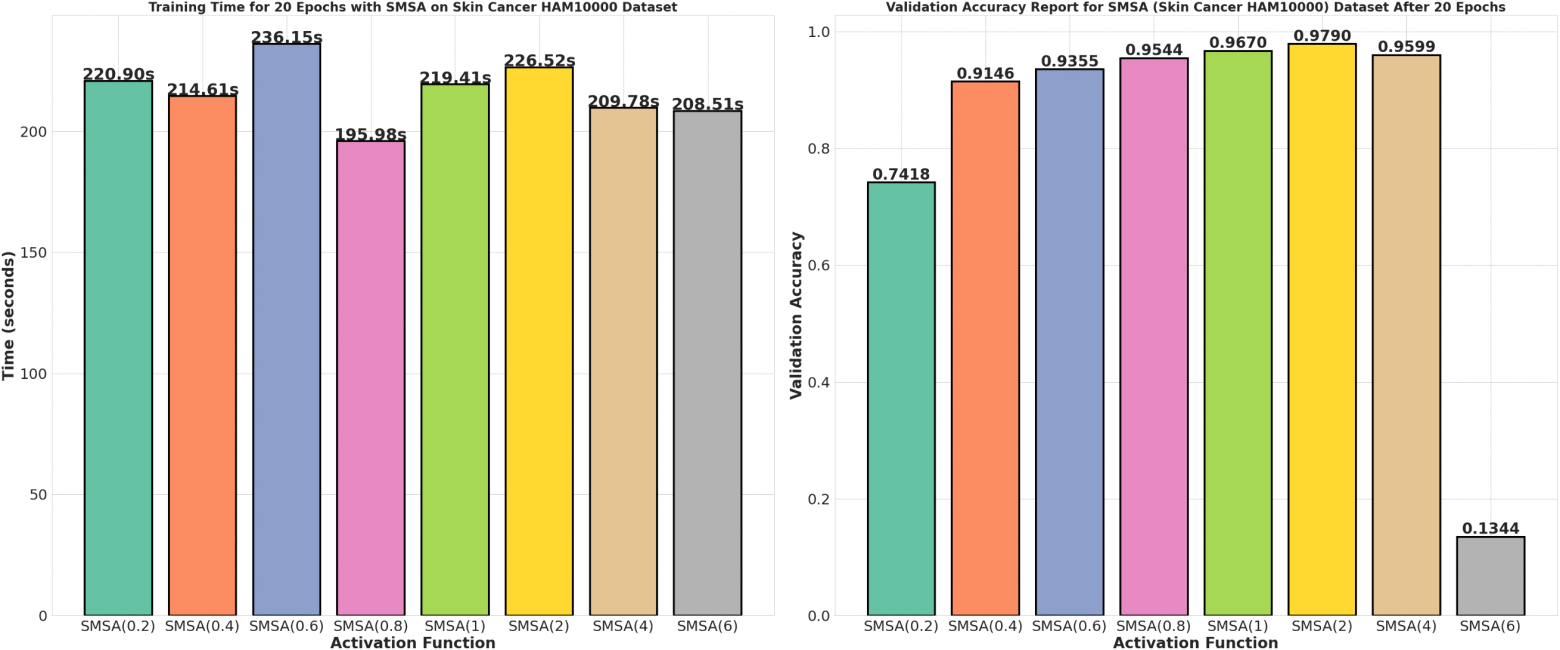
Effect of the SMSA scaling parameter (*n*) on convergence accuracy and training duration of the optimized CNN model on the HAM10000 dataset.

**Fig 11:**
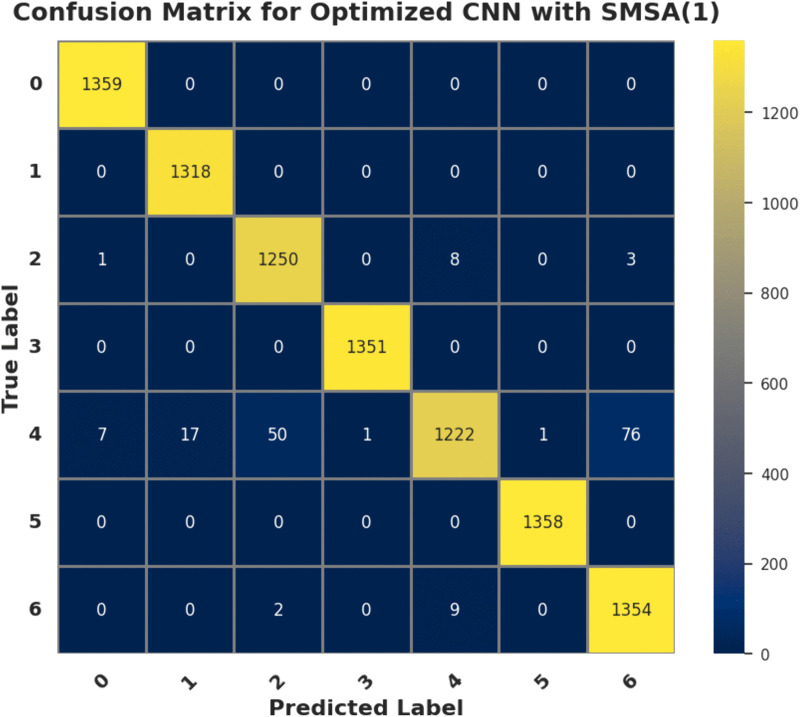
Effect of the SMSA scaling parameter (*n*) on the confusion matrix of the optimized CNN model for the HAM10000 dataset.

**Fig 12:**
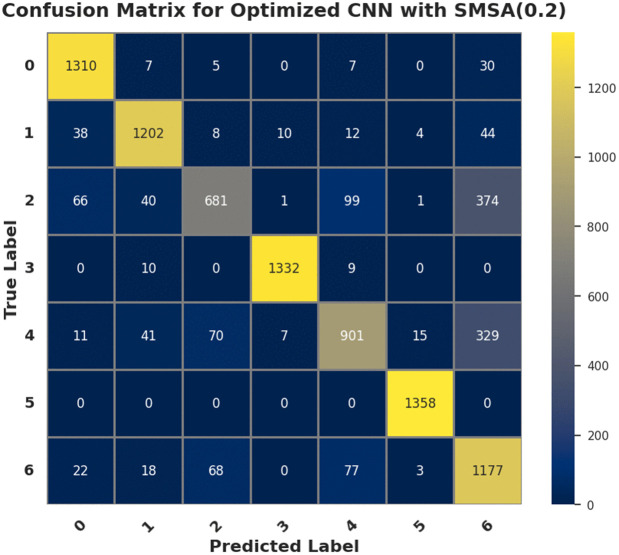
Effect of the SMSA scaling parameter (*n*) on the confusion matrix of the optimized CNN model for the HAM10000 dataset.

**Fig 13:**
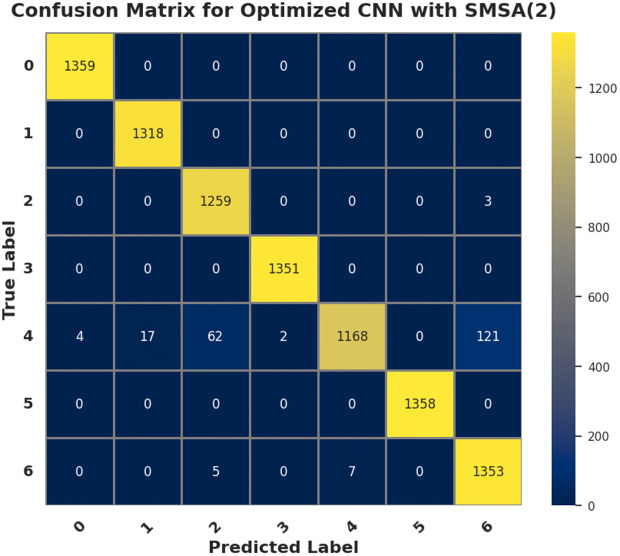
Effect of the SMSA scaling parameter (*n*) on the confusion matrix of the optimized CNN model for the HAM10000 dataset.

**Fig 14:**
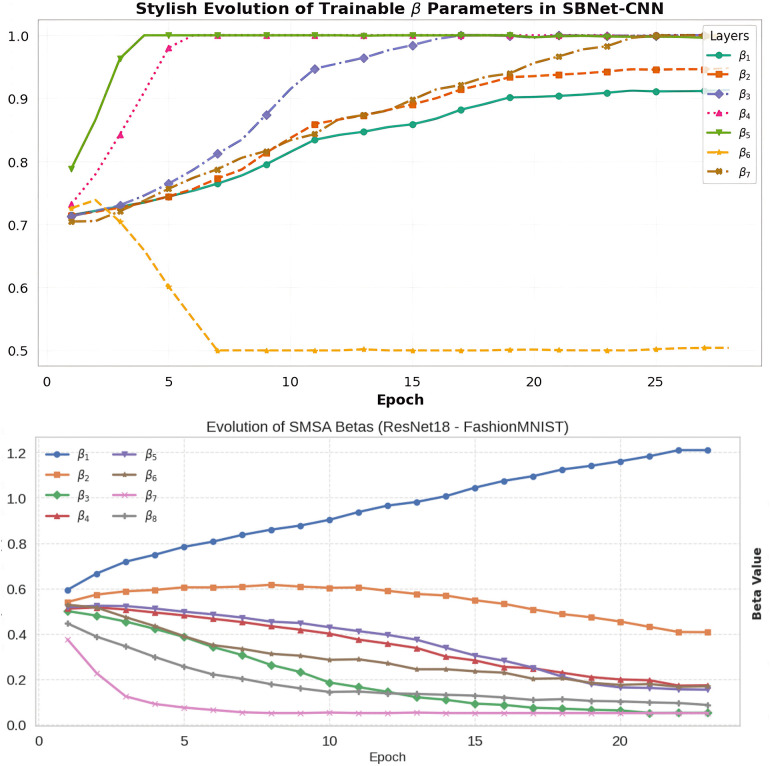
Layer-wise trajectory of the trainable slope parameter (β) in the SMSA(x,β) activation function for ResNet18 and SBNet-CNN on the Fashion-MNIST dataset.

**Fig 15:**
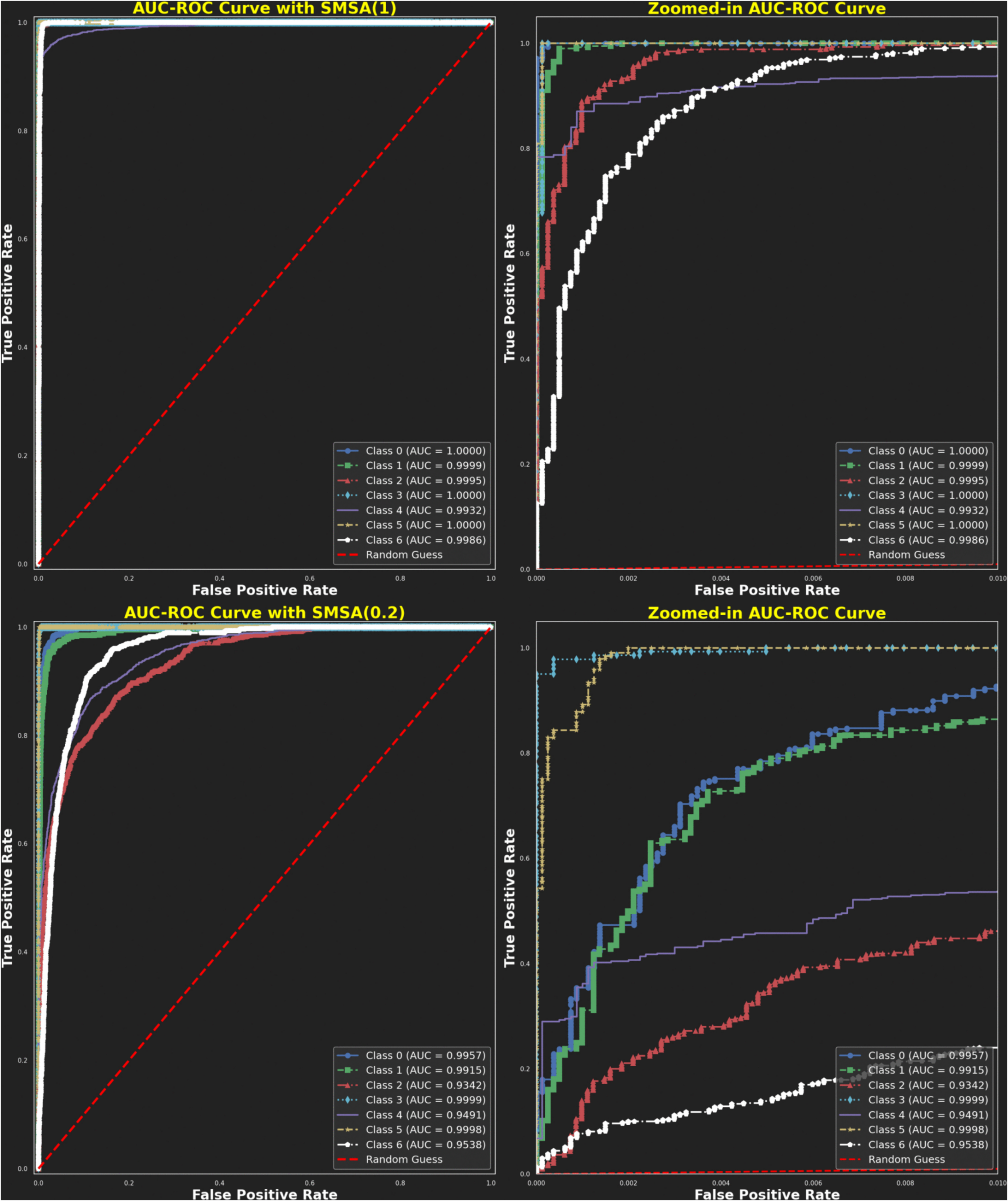
Effect of the SMSA scaling parameter (*n*) on the area under the curve (AUC) of the optimized CNN model on the HAM10000 dataset.

**Fig 16:**
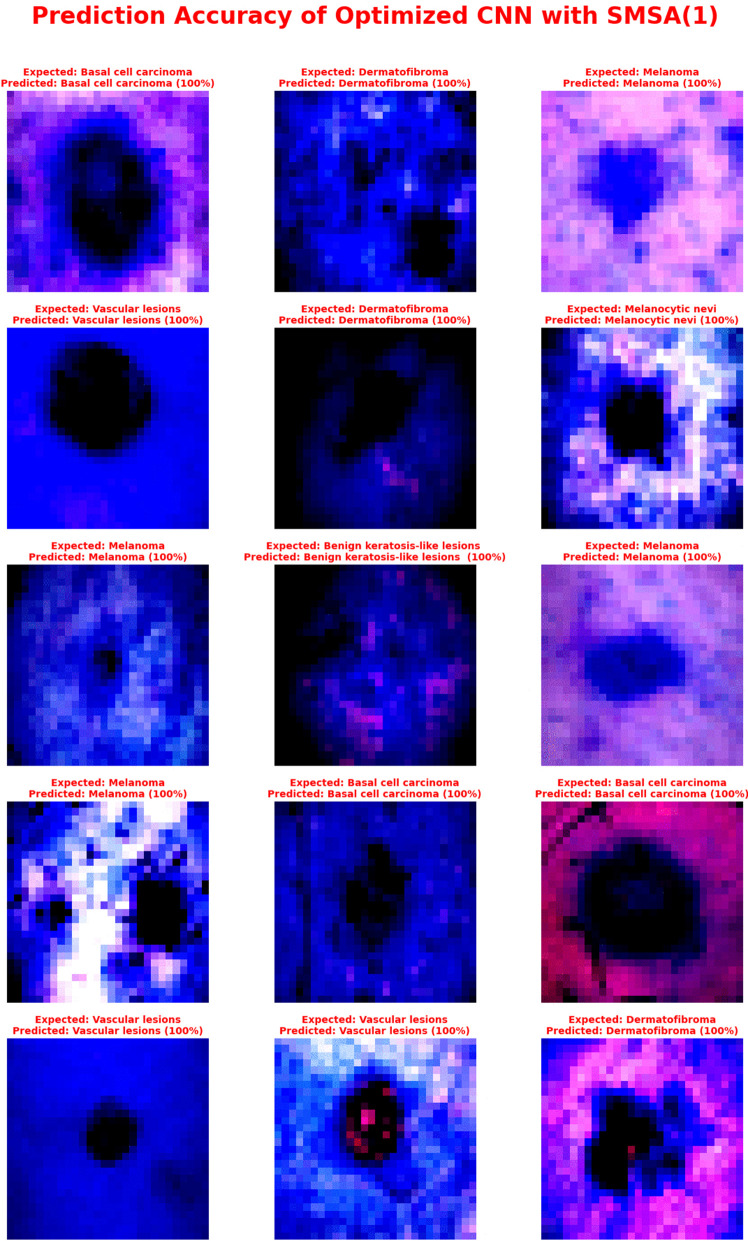
Prediction accuracy of the optimized CNN model using the SMSA activation function with *n* = 1.

**Fig 17:**
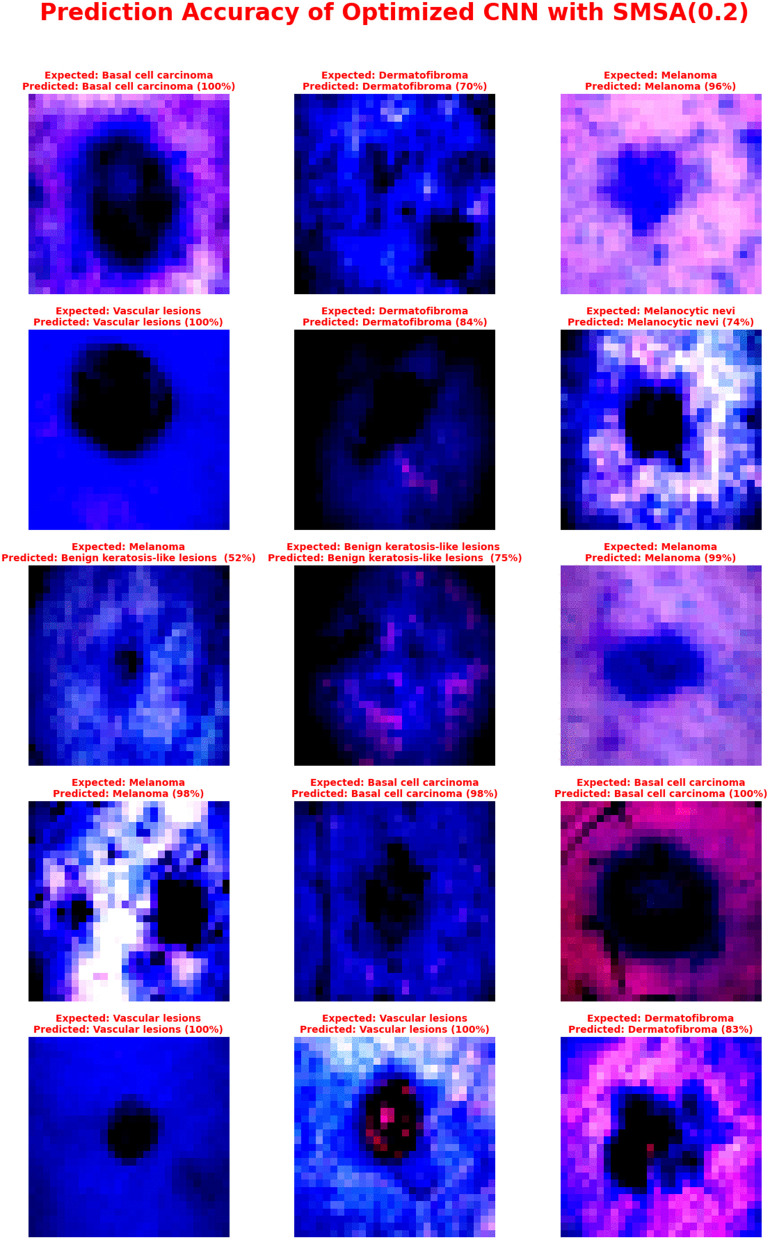
Prediction accuracy of the optimized CNN model using the SMSA activation function with *n* = 0.2.

**Table 10:** Optimal SMSA slope parameter (*n*) ranges and observed performance behavior across different datasets.

Dataset	Best Range of *n*	Key Behavior
MNIST	0.6–10.0	Fast convergence, reduced misclassification, and stable gradient propagation.
Fashion-MNIST	0.6–2.0	Improved accuracy, accelerated learning, and strong generalization performance.
HAM10000	0.8–2.0	High classification accuracy, smooth gradient decay, and robust feature discrimination.

For the MNIST dataset, Figs 4 and 5 and Tables 11 and 12 have shown that the parameter *n* possesses a broad optimal range from 0.6 to 10, within which convergence has remained rapid and gradient stability has been preserved. However, excessive gradient exploitation at lower values of *n* has marginally affected generalization. When the convolutional neural network is used with low values of n, it is highly likely to overfit, Memorizing the details of the training images instead of capturing generalised, clustering properties that define classes. As such, the model achieved high training accuracy but not be able to maintain performance when seen on unseen data, which leads to a significantly lower validation accuracy than the training accuracy, although overall classification performance has remained robust within this interval.

**Table 11:** Training and validation loss and accuracy obtained using the SMSA activation function with different scaling parameter values (*n*) on the MNIST dataset.

Activation Function	Training Loss	Validation Loss	Training Accuracy	Validation Accuracy
SMSA(0.2)	0.0567	0.0315	0.9821	0.9895
SMSA(0.4)	0.0360	0.0290	0.9880	0.9904
SMSA(0.6)	0.0314	0.0227	0.9901	0.9921
SMSA(0.8)	0.0292	0.0188	0.9902	0.9952
SMSA(1.0)	0.0275	0.0250	0.9908	0.9936

**Table 12:** Training and validation loss and accuracy obtained using higher values of the SMSA scaling parameter (n) on the MNIST dataset.

Activation Function	Training Loss	Validation Loss	Training Accuracy	Validation Accuracy
SMSA(2)	0.0232	0.0197	0.9920	0.9944
SMSA(4)	0.0296	0.0260	0.9903	0.9912
SMSA(6)	0.0161	0.0174	0.9958	0.9954
SMSA(8)	0.0187	0.0198	0.9937	0.9941
SMSA(10)	0.0165	0.0161	0.9965	0.9969

In the case of the Fashion MNIST dataset, Figs 6 and 7 and Tables 13 and 14 have shown that the optimal range has significantly narrowed to 0.6−−2.0. Within this interval, the model has achieved accelerated learning and enhanced generalization. At very small values of *n*, gradient exploitation does not accelerate convergence resulting in a decline in predictive accuracy. In contrast, values of *n* that are too large trigger a slowing of the convergence process that does not offer further generalization performance improvements, and hence, a dataset-specific trade-off is indicated. The observations highlight the fact that the SMSA (Eq (1)) mechanism is highly sensitive to the scaling parameter. As a result, it has made careful calibration of this parameter crucial to the maintenance of stability in the performance conditions of a variety of learning situations. HAM10000 collection is an interesting case study of high-dimensional classification literature, with seven clinically important dermatoscopic classes, which have a significant level of overlap of features.

**Table 13:** Training and validation loss and accuracy obtained using the SMSA activation function and baseline activation functions on the Fashion-MNIST dataset.

Activation Function	Training Loss	Validation Loss	Training Accuracy	Validation Accuracy
SMSA(2)	0.2354	0.2329	0.9129	0.9149
SMSA(4)	0.2145	0.2140	0.9276	0.9219
SMSA(6)	0.2097	0.2094	0.9278	0.9280
SMSA(8)	0.2650	0.2561	0.9061	0.9061
SMSA(10)	0.2725	0.2761	0.9042	0.9030
ReLU	0.2499	0.2285	0.9105	0.9206
Leaky ReLU	0.2765	0.2520	0.9001	0.9105
ELU	0.2600	0.2434	0.9062	0.9109

**Table 14:** Training and validation loss and accuracy obtained using different values of the SMSA scaling parameter (*n*) on the Fashion-MNIST dataset.

Activation Function	Training Loss	Validation Loss	Training Accuracy	Validation Accuracy
SMSA(0.2)	0.3233	0.2739	0.8842	0.9003
SMSA(0.4)	0.2548	0.2438	0.9074	0.9120
SMSA(0.6)	0.2019	0.2014	0.9223	0.9222
SMSA(0.8)	0.2006	0.2002	0.9311	0.9313
SMSA(1.0)	0.2009	0.2008	0.9285	0.9287

A carefully fine-tuned convolutional neural network, used in combination with SMSA (Eq (1)), has converging training and validation accuracy curves, especially when *n* is in the range of 0.8 to 2.0. In this interval, CNN indicated high generalization and opposed to memorization, thus demonstrating that SMSA supports a nonlinear approximation of the complex class structure. In the case of the HAM10000 dataset, Table 15, Figs 8–10 illustrate that the optimal results of parameter tuning lie between the range of 0.8 and 0.2. The model is always characterized by the gradual degradation of its gradient, optimal convergence, and high classification rates within this range. Besides, the differences in *n* within this spectrum increase robustness to the inherent visual similarities of the dataset, which also maintains generalisation without undermining convergence dynamics. The empirical evidence supports the formulated hypothesis that the slope-control parameter acts as a key modulator of gradient dynamics, and thus, enables adaptive control of the convergence rate, the computational costs, and the generalization ability overall of the model to a wide range of complexities of data-sets. Also, the CNN is stable in terms of training over large epochs, without the tendency of gradient vanishing or over-fitting. The behaviour of this nature highlights the effectiveness of the SMSA, represented as in Eq (1), in trade-off representational power with computational efficiency. The influence of the SMSA function’s slope parameter in Eq (1) on the optimization performance of optimized CNN has been systematically evaluated using the HAM10000 skin lesion dataset. Extensive experimentation has revealed that low values (0–0.3) have caused sparse and unstable gradient propagation, resulting in ineffective feature learning and poor classification accuracy, as illustrated in Figs 6, 8 and 9. The key findings are that a big disjuration between training and validation accuracy suggests that the convolutional neural network is mainly memorizing data peculiarities rather than generalising. Conversely, overlapping training and validation curves indicate that the network has acquired abstract transferable features. In addition, the performance of classification is related to the parameter n, the slope of activation. Typically, neural network models with moderate number of parameters give good performance with particular data sets of different complexities.

**Table 15:** Training and validation loss and accuracy obtained using the SMSA activation function on the skin cancer dataset with an optimized CNN trained for 20 epochs.

Activation Function	Training Loss	Validation Loss	Training Accuracy (%)	Validation Accuracy (%)
SMSA(0.2)	0.8762	0.7002	66.57	73.68
SMSA(0.4)	0.3348	0.2572	88.52	90.97
SMSA(0.6)	0.2350	0.1638	92.21	94.45
SMSA(0.8)	0.1575	0.1314	94.71	95.60
SMSA(1.0)	0.0902	0.0885	96.69	97.04
SMSA(2.0)	0.0776	0.0726	97.39	97.90
SMSA(4.0)	0.1442	0.1325	95.03	95.99
SMSA(6.0)	1.9460	1.9462	14.15	13.44

Moreover, the AUC scores in this range have remained consistently low, with significant class overlap and indistinct decision boundaries, as evidenced by Fig 15. The confusion matrix in Figs 11–13 has further confirmed elevated misclassification rates due to inadequate feature separability. Figs 16 and 17 have demonstrated that the slope parameters have significantly improved the class approximation confidence levels in the optimized CNN. It has also been noted that the network has been more prone to memorize features instead of learning abstract representations in the absence of proper slope control because of over exploration of gradient. This has been the main cause of its continually low levels of confidence on the prediction of classes. The confusion matrix provides a class based perspective of the model performance by indicating the classes the model is classifying well and those that are difficult due to overlapping features. It points out where the model has difficulties with making clear distinctions. The SMSA(x,n) activation gets better separation of the classes by controlling the gradient using slope parameter n to give finer decision boundary between the problematic classes.

Conversely, medium slope values (about *n* = 1–4) have facilitated steady gradient flow which has facilitated better learning of representations. This has been translated to sharper class separation, better AUC in Fig 15 and better classification reliability. The curves of ROC of this regime have proved to be more sensitive and specific and this also swayed toward better suitability of these *n* values. The findings show that slope modulation can be significant to specify network dynamics and to optimize decision boundaries to complex visual recognition tasks such as skin lesion classification. On the other hand, a moderate range of slope (0.6–2.0) has supported strong and steady gradient flow that can ensure the CNN converges effectively and has higher test accuracy and significantly better AUC based on all classes. The confusion matrix in Figs 11–13 in this optimum range (0.6–2.0) has recorded clear and well separated boundaries of classes with minimum errors in classification. Nevertheless, in cases where the slope values have been overdone (3–10 and above), gradient saturation has already been experienced and the gradients have vanished around zero indicating the eventual stoppage of learning. This has caused stagnated network optimization, leveled accuracy and deterioration in the AUC performance. The results indicate that adaptive slope modulation inside SMSA Eq (1) is essential to the control of the learning dynamics of deep CNNs, particularly on complex data such as HAM10000, where accuracy maximization, increasing AUC values, and reducing errors on the confusion matrix are essential and discriminative classification results are found.

The adaptive evolution of the β parameter across multiple layers, as guided by SMSA with Eq (2), has been effectively illustrated in Fig 14 for SBNet-CNN and ResNet18 on the FashionMNIST dataset, Fig 19 for VGG16-CNN on FashionMNIST, and Fig 26 for Optimiz-CNN on HAM10000. This behavior has contributed significantly to achieving optimal convergence and enhancing classification performance across diverse architectures and datasets.

**Fig 18:**
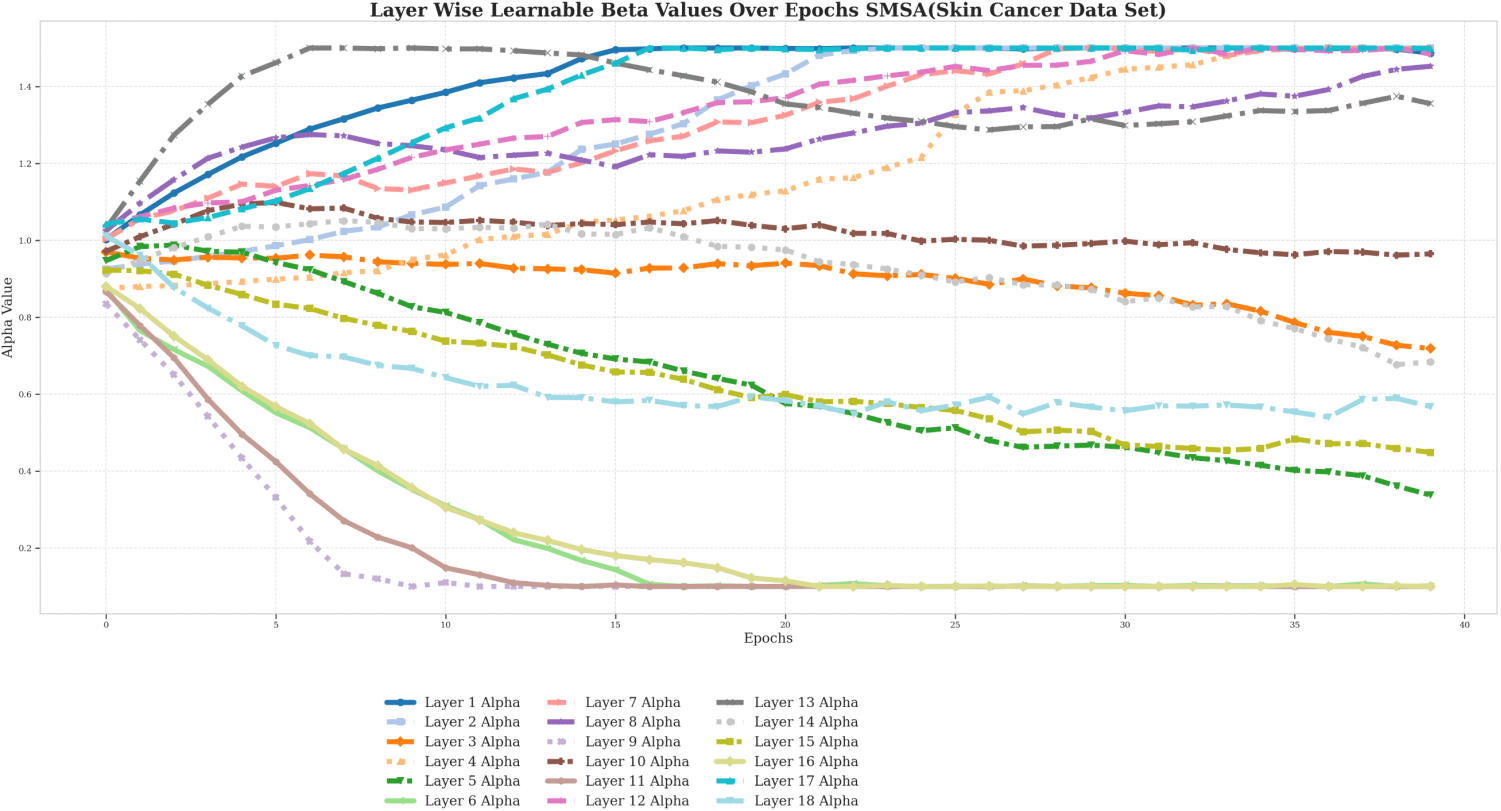
Layer-wise evolution of the adaptive slope parameter (β) in the SMSA activation function on the HAM10000 skin cancer dataset.

**Fig 19:**
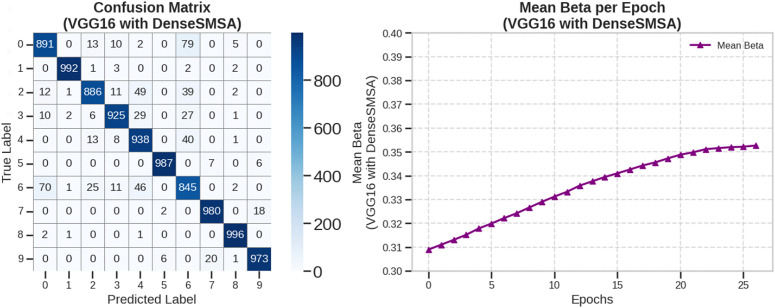
Confusion matrix and corresponding average layer-wise β values demonstrating the adaptive behavior of the SMSA activation function in the VGG16-CNN architecture on the Fashion-MNIST dataset.

A detailed study of the β parameter curves of the layers of SBNet-CNN has shown that in about 90% of the layers, the β value has been constantly within the 0.6 to 1 range as shown in Fig 14. This trend has indicated the adaptive behaviour of the adaptive Eq (2) with Eq (12) mechanism that has provided the necessary non-linearity to the SBNet-CNN architecture. In this β regime, the denominator in the adaptive SMSA(x,β) has been large enough to dominate the numerator term in the adaptive SMSA formulation, leading to a suppression of gradient exploration in most of the layers, which are seen in Fig 2. This suppression has not caused the network to merely memorize the training image features; rather, it has facilitated the extraction of generalized feature representations, leading to the formation of well-separated and distinct decision boundaries. Consequently, SBNet-CNN has achieved higher test accuracy and robust generalization performance, as substantiated by the results presented in Fig 21 for SBNet-CNN. In contrast, the β trajectories observed in ResNet-18 have shown a continuous decline, where most β values have decreased from 0.4 down to 0.01 as in Fig 14. In this lower β regime, the numerator term of adaptive SMSA has become dominant, which has intensified gradient exploration in approximately 93% of ResNet-18’s layers in Fig 14 and mostly layer having non linearity as in Fig 2.

This excessive exploration has led the network towards memorization of training-specific features, resulting in overlapping and ambiguous decision boundaries between various image classes. Consequently, misclassification rates have increased, and a substantial performance gap has emerged between validation and training accuracies, which has been consistently observed in Fig 21 for ResNet18-CNN. For VGG-16, instead of analyzing each layer’s β trajectory individually, an average β trajectory across layers has been computed. These averaged values have predominantly remained within the range of 0.3 to 0.36 obserserved in Fig 19, suggesting that a moderate degree of gradient exploration has been maintained less aggressive than ResNet-18 but higher than SBNet-CNN. This trend has been captured in the apparent differences in training, validation, and test accuracies of VGG-16 performance measures. HAM1000 classification task has brought more challenging issues because it is characterized by image classes sharing high visual similarities. Here, it can be seen that the non-linear activation pattern of the network has shown the network use of a unique behavior of the network as denoted by its adaptivity behavior known as its β.

This observation has revealed the ability of the adaptive SMSA with the mechanism of Eq (2) in conjunction with the one of Eq (12) to adapt its responses with changing image difficulty in order to enable effective learning and enhanced classification of images under difficult conditions. The change in the values of β in all the layers of the optimized CNN has been depicted in Fig 18. Most layers have ensured that their β is kept between 0.4 and 1, and this has contributed to reduction of the over-exploration of the gradient. A few layers have been observed to be close to 0.1, with a localized area of high gradient activity. In general, non-linearity in behavior between layers has been noticed to be consistent as shown in Fig 18. The performance results have been verified by the result of Fig 26, wherein obtained rapid convergence, convergence stability and great generalization. The complementary analysis of Figs 18 and 26 provides a result with significant scientific value. The orientation of the learnable parameter β is highly divergent within the network layers, most of the values taking values over 0.4.

The implication of such trend is the existence of an active, layer-specific adaptivity, as opposed to a relaxation to some fixed (or even saturated) regime of activation. Thereby, the observed variability supports the idea that the Adaptive SMSA activation is not breaking down into a single and tight functional archetype, rather, each of the layers modulates its nonlinearity independently according to its representational demands.

This dynamics are evidence of an effective, layer-by-layer exploration of the activation space wherein different network depths learn to be differentially sensitized to curvature which in turn allows learning of hierarchical representations of features. Fig 26 also showed that no observable discrepancy was found between training and validation accuracy, and test accuracy is highly correlated with training and validation accuracy. The implication of such concordance is that there is little overfitting and strong extrapolating extracted features. The consistency in training, validation, and testing demonstrates that the model has acquired consistent and stable characteristics. This signifies that the results are not a result of overfitting but consistant learning across all the stages. Although the adaptive SMSA mechanism helps to control the non linearity modulation, a large share of the observed generalization performance can be attributed to the O -CNN architecture, which effectively exploits adaptive activation dynamics. It has been empirically demonstrated that convolutional neural networks perform better with adaptive nonlinearity added. It increases the learning capability of the networks and leads to a more stable optimization process, it improves generalization. As one looks at the learnable parameter β which gets successively learnt by different layers there is a definable stratification of the functions in the network. Initial layers of the convolutional model store relatively small maxima of curvature, and thus facilitate the effective extraction of low-level spatial information (edges and textures). More so, successively deeper layers assume increasingly larger values of beta, which simplifies more powerful nonlinear transformations, which are needed to formulate complex semantic representations. This slope of the gradient of β is harmonious with the canonical representation learning hierarchies of canonical hierarchical representation, in which the complexity of features increases in parallel with the depth of networks. Besides, no parameter saturation ensures a constant gradient flow during training, effectively preventing the issue of vanishing or exploding gradients and hence a smooth convergence. Formulation SMSA(x, n) means that the slope parameter is to be determined manually, which is more engineering work, and potentially not as scalable to a wide variety of tasks. This formulation was used to analyze the behavior of curvature and gradient characteristics that depend on the interval of such an activation function. To overcome this limitation, different versions of adaptive variants such as (x, β) and layer-wise learnable parameters were introduced that allows the network to autoregulate the parameters of activation during training. It is an autonomous process which improves optimization stability, scaling to large datasets, and transfer to diverse datasets and CNN architectures. SMSA(x, β) in which the parameter β is trained end-to-end by back-propagation. As a result, the adaptive one allows the data-driven gradient changes, which enhances the scalability and cross-task generalisation without having to adjust any parameter manually.

### Performance comparison with baseline activation functions

**Fig 20:**
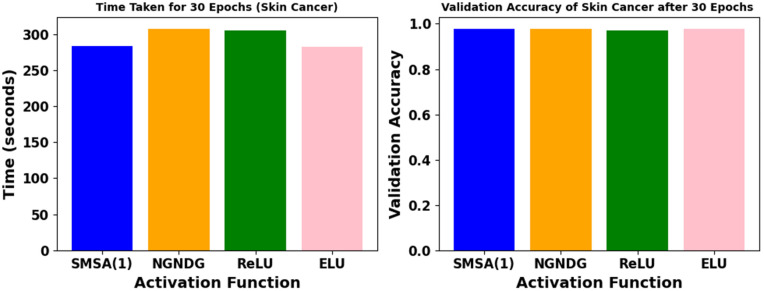
Confusion matrix and average layerwise β values illustrating the adaptivity of the adaptive SMSA activation function on the FashionMNIST dataset using the VGG16-CNN architecture.

The FashionMNIST dataset with its similarity among the inter-classes and minimal differences in features have posed a serious challenges in the design of the activation functions. Tables 16–19 and Fig 20 have given a comparative analysis of the standard and advanced activations, the suggested SMSA (both fixed and adaptive). The fixed SMSA specified by Eq (1) has demonstrated a higher validation accuracy than ReLU, ELU, and Leaky ReLU with a high of 93.5% accuracy in Table 16. This has been enhanced by the fact that it has a symmetric structure and an adjustable slope in the form of stabilized gradient flow and quick convergence despite overlapping classes. Standard static activations have pushed most of the layers into zero gradient or unstable areas and cannot thus be optimized. Conversely, SBNetCNN with the adaptive SMSA in the layer-wise in Eq (2) with Eq (12) has the highest test accuracy of 97.52% according to Table 17.

**Table 16:** Performance comparison of ResNet-18 using different activation functions.

Activation Function	Precision	Recall	F1 Score	Test Accuracy
RTA [22]	0.8307	0.7885	0.7648	0.7885
ReLU [22]	0.8252	0.7644	0.7300	0.7644
Tanh [22]	0.1421	0.7516	0.7126	0.7516
Swish [22]	0.8206	0.7484	0.7060	0.7484
ELU [22]	0.8034	0.7131	0.6511	0.7131
Proposed SMSA in Eq (2)	**0.9401**	**0.9397**	**0.9409**	**0.9402**

**Table 17:** Performance and Stability Analysis of Activation Functions on Fashion MNIST.

Activation Function	Test Accuracy	Computational Cost	Convergence	Loss Stability	CNN Model
ReLU	90.30%	Low	Fast (Fluctuates)	Unstable	Custom CNN
ELU	91.40%	Medium	Fast	Stable	Custom CNN
Leaky ReLU	91.46%	Medium	Fast	Good Stability	Custom CNN
Swish	91.60%	High	Smooth	Very Stable	Custom CNN
Mish	91.65%	High	Consistent	Highly Stable	Custom CNN
Proposed SMSA in Eq (1)	92–93.50%	Low (Tunable)	Fast and Consistent	Stable	Custom CNN
Proposed SMSA in Eq (2)	94.00%	Low (Tunable)	Fast and Consistent	Stable	ResNet18
AHerfReLU [21]	93.93%	High	Consistent	Stable	Custom CNN
Swish [32]	94.10±0.34%	Moderate	Consistent	Stable	HQCC
Proposed SMSA in Eq (2)	97.52%	Low (Tunable)	Fast and Consistent	Best Stability	SBNet

**Table 18:** Performance Comparison of Different Activation Functions on the Skin Cancer Dataset.

Activation Function	Training Loss	Validation Loss	Training Accuracy	Validation Accuracy	Training Time (s)
ReLU	0.0579	0.0767	98.20%	97.72%	307.55
ELU	0.0478	0.0750	98.41%	97.81%	283.29
NGNDG	0.0535	0.0752	98.22%	97.00%	304.75
**Proposed SMSA**(1)	**0.0393**	**0.0745**	**98.73%**	**97.87%**	**281.81**
**Proposed SMSA**(2)	**0.0393**	**0.0745**	**99.1%**	**98.9%**	**210.25**

**Table 19:** Overall performance summary of the proposed CNN architecture with SMSA activation on the Skin Cancer (HAM10000) dataset. The model achieves near-perfect classification metrics with stable training behavior.

Metric	Accuracy	Recall	Precision	F1-score	AUC	Epochs
**Train**	0.991	0.991	0.990	0.990	0.9996	37
**Validation**	0.989	0.990	0.988	0.989	0.9994	37

**Fig 21:**
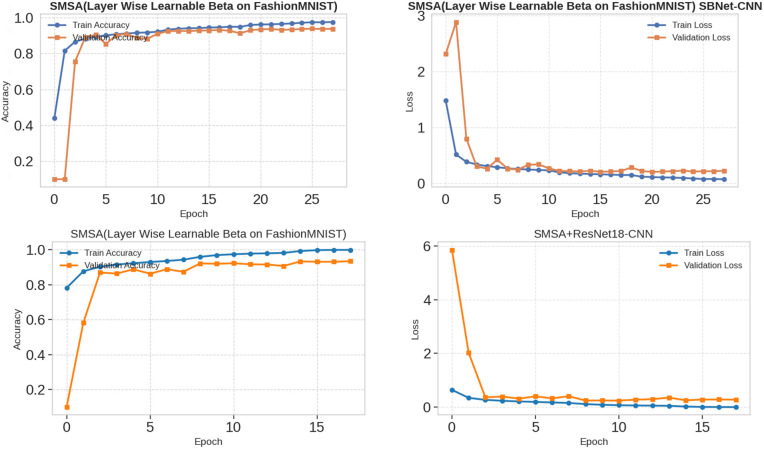
Training and validation accuracy/loss comparison of SBNet-CNN and ResNet18-CNN with layer-wise adaptive SMSA activation on the FashionMNIST data set.

It has been able to defend close to 95% of layers against gradient problems by training a discrete nonlinearity on each layer. This flexibility has introduced better boundaries in classes, generalization and performance even with complicated overlapping conditions. SMSA has provided better gradient control and faster convergence at a lower cost than functions such as Mish, Swish, AHerfReLU [21] and swish [32]. These findings have provided adaptive SMSA in Eq (2) with Eq (12) as an effective and valid strategy of classification of image rich in features. The effectiveness of the layer-wise adaptive SMSA activation function has been proven by the results provided in Table 17, together with the evaluation measures of ResNet18. In contrast to older nonlinearity: ReLU, Swish, ELU and more recent ones like RTA, the adaptive SMSA in Eq (2) with Eq (12) has allowed every convolutional layer to learn independently its best nonlinearity in training. This flexibility has enhanced learning processes, increased expressive capacity and led to improved classification performance. When taking the example of ResNet18, which combines more than 30 pretrained layers, the adaptive SMSA has been implemented across around 70% of the network layers. This partial integration has been found to be enough not to degrade gradient in most parts of the network leading to the reported gains in performance in all the major performance measures such as precision, recall, F1-score as well as test accuracy on the FashionMNIST dataset. Nevertheless, the gradient flow in ResNet18 and Vgg16 has sometimes become more pronounced due to the fixedness of the remaining pretrained layers (around 30%), and it prevents full removal of overlapping decision boundaries between the models. Therefore, despite ResNet18 with adaptive SMSA being superior in all performance measures compared to all other static and recent activation functions, it has been shown to have slightly low classification accuracy compared to SBNet-CNN. This distinction is accredited to the absolute adaptability of SBNet-CNN, that has allowed more considerable movement of gradients and enhanced capacity to discriminate features across all levels. However, the adaptive SMSA as expressed in Eq (2) along with the expression in Eq (12) has proven itself to be a well-developed and scalable method in achieving better CNN performance, especially in deep networks such as ResNet18. To ensure a fair and rigorous evaluation, all activation functions have been implemented using the same optimized CNN architecture under identical environmental and hyperparameter settings on the HAM10000 (skin cancer) dataset. The training regime, data preprocessing pipeline, learning rate schedule, and optimizer configurations have been uniformly maintained across experiments. A critical analysis of Fig 21 shows that although both SBNet and ResNet-18 show a perceptible gap in training and validation accuracy, the gap is significantly bigger in the case of ResNet-18. The β trajectories for ResNet18 in Fig 14 point to the fact that, in ResNet-18, the maintains the value of beta much less than 0.3 (Exploration occure), and not having variation in the β trajectories for layers after epoch 15(adaptivity vanished). On the other hand, SBNet maintains the value of β much higher than 0.6, thus allowing continuous layer-wise curvature modulation and stabilization, which together provide a contracted feature generalization and proven better out-of-sample performance.

Fig 20 and Table 18 have presented a clear performance differentiation among the tested activation functions, namely ReLU, ELU, NGNDG, and the proposed SMSA. Notably, SMSA in its fixed form (*n* = 1) has already achieved the highest validation accuracy (97.87%) and the lowest training time (281.81s) among non-adaptive methods, surpassing even recent alternatives like NGNDG and ELU in terms of both convergence speed and generalization. Moreover, training and validation loss has been steady and low in case of SMSA which shows a stable and efficient learning curve. Performance has further been increased with the adaptive version of SMSA, which is controlled by the Eq (2). Table 19 shows that this setup has obtained close to perfect classification scores: 98.9% validation accuracy, 0.989 F1-score and a very high AUC of 0.9994 which indicates that the model is very sensitive discriminatively. Also, the model has taken just 37 epochs before converging, which further shows its efficiency in computing. The gradient flow, feature separability and stability of the loss landscapes have been improved by the capability of the adaptive SMSA to dynamically change its slope across layers. This has been especially useful with the HAM10000 dataset that consists of highly imbalanced and morphologically overlapping skin lesion classes. This series of findings have all affirmed that the suggested SMSA activation particularly in its adaptive form has significant benefits compared to traditional and recent activation functions in terms of the efficiency and effectiveness of training, as well as the classification.

### Comparative performance analysis of CNN models

This subsection has provided a comparative study of CNN model performances. This assessment has been performed on the Fashion MNIST dataset, and then on the HAM10000 dataset. An in-depth comparison of the results obtained on the two datasets is presented.

#### Comparative evaluation of SBNet-CNN with ResNet18 and VGG16 on Fashion-MNIST.

To confirm the proficiency of the suggested SBNet-CNN architecture in Tables 2–4 that had been combined with layer-wise adaptive SMSA activations given in Eq (2), a thorough comparison with well-known CNN architectures, namely ResNet18 and VGG16, was performed. This analysis has highlighted architectural performance, number of parameters, computational performance, and classification performance on the Fashion-MNIST data.

**Fig 22:**
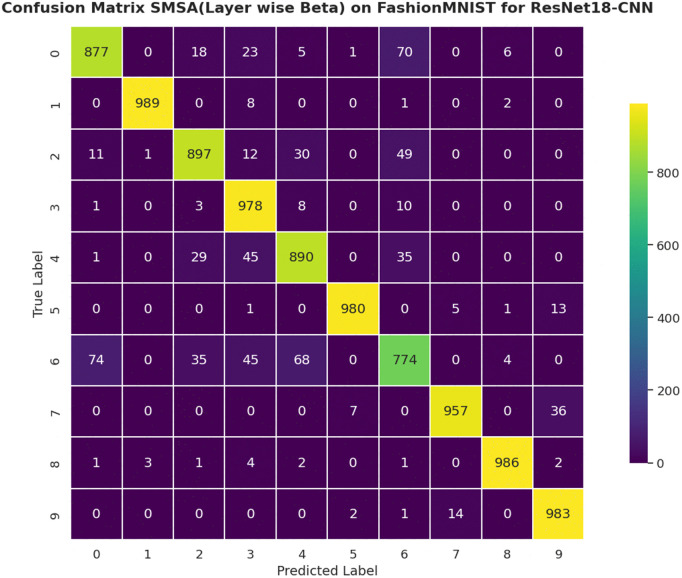
Confusion matrix comparison of SBNet-CNN and ResNet18-CNN with layer-wise adaptive SMSA activation on FashionMNIST.

**Fig 23:**
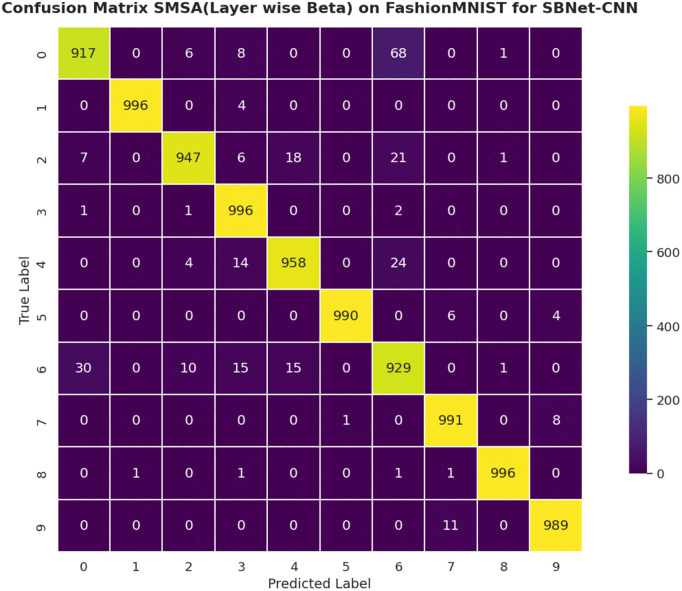
Confusion matrix comparison of SBNet-CNN and ResNet18-CNN with layer-wise adaptive SMSA activation on FashionMNIST.

**Fig 24:**
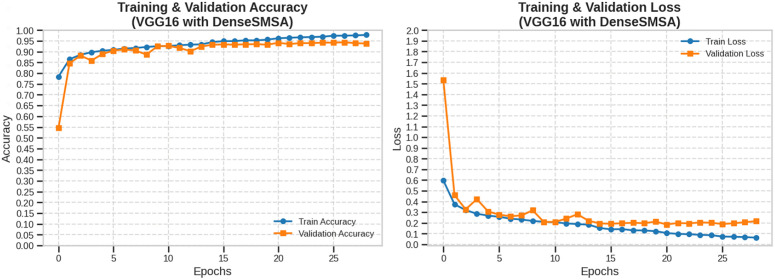
Training and validation accuracy/loss comparison of Vgg16-CNN with layer-wise adaptive SMSA activation on the FashionMNIST data set.

**Fig 25:**
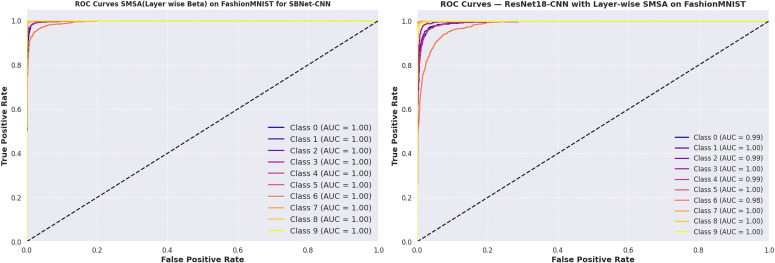
AUC comparison of SBNet-CNN and ResNet18-CNN with adaptive SMSA on FashionMNIST.

In experiments with layer wise adaptive SMSA for Fashion Mnist, ResNet18 and VGG16 have been augmented with adaptive SMSA activations to ensure a fair performance comparison. While these architectures have benefited from the learnable non-linearity introduced by SMSA, their inherently deep and parameter-heavy designs have still resulted in higher computational costs. In contrast, the proposed SBNet-CNN has adopted a lightweight architectural design that, combined with layer-wise adaptive SMSA activations, has allowed the network to flexibly modulate feature transformations while maintaining a minimal parameter footprint. Specifically, SBNet-CNN (with SMSA) has comprised only **1.7 million parameters**, significantly fewer than the **11.2 million** and **14.7 million** parameters of ResNet18 and VGG16, respectively. Consequently, SBNet-CNN has achieved over an **80% reduction in parameter count** compared to VGG16, while simultaneously enhancing model expressiveness through SMSA-induced slope modulation at each layer. Fashion-MNIST dataset has presented a much more difficult classification problem than traditional benchmarks as it is more intra-class variant, less inter-class separability, complex textures and shapes, grayscale fine-grained variability, and low image resolution (28 x 28 pixels) per image. Delicate differences among visual similar classes like shirts, tops, and coats have required fine feature extraction abilities, which traditional architectures have found difficult to satisfy. Also, the lack of the color information has forced models to use only the patterns of structure, and strong non-linear feature transformations have become essential in the non-linear classification. It has been rigorously demonstrated that the proposed SBNet-CNN is indeed effective in dealing with these complexities through its lightweight yet flexible architecture using its evaluation on this dataset. Combining layer-wise adaptive SMSA activations has allowed the network to non-linearly, slope, and activation range control per layer, allowing it better resolution of finer spatial detail with minimal computational cost. The results of experiments in Tables 20–Table 21 were also confirmed by performance curves demonstrated in Figs 21–25. The SBNet-CNN has reached a validation accuracy of **98.2%** and a training accuracy of **99.1%**, and it only takes **12 epochs** to converge.

**Table 20:** Compact Comparison of SMSA-based CNN Architectures on FashionMNIST.

Model	Train Accuracy (%)	Validation Accuracy (%)	Parameters (M)	Epochs	Gap (%)
SBNet-CNN (SMSA)	99.1	98.2	1.9	12	1.0
ResNet18 (SMSA)	99.5	94.5	11.2	16	5.0
VGG16 (SMSA)	99.2	95.0	14.7	18	4.2

**Table 21:** Performance Comparison of SBNet-CNN with ResNet18 and VGG16 on Fashion-MNIST.

Model	Test Accuracy (%)	Epochs to Converge	Training Time (s)	FLOPs (Millions)
**SBNet-CNN (SMSA)**	**97.52**	**12**	**630**	**320**
ResNet18	94.18	16	910	1800
VGG16	94.13	18	1025	2200

Comparatively, ResNet18 with SMSA took only **16 epochs** to converge and the validation accuracy was only **94.5%** compared to**99.2%** with ResNet18, even though the training accuracy was a bit higher. Equally, VGG16 combined with SMSA converged after **18 epochs** and reached a validation accuracy of **95.0%**, and training accuracy of **99.5%** and **99.1%** indicating a more significant generalization gap. These findings indicate that the adaptive SMSA-based SBNet-CNN is computationally cost-effective, and has a substantially better generalization capacity and learning throughput than the parameter-intensive traditional architectures.

Confusion matrices of SBNet-CNN, ResNet18-CNN and Vgg16-CNN on the Fashion-MNIST test data set have been generated to assess classification quality as depicted in Fig 19 and Figs 22 and 23. SBNet-CNN has its overall accuracy of **97.52%** (9752 correct and 248 incorrect) which compares to the accuracy of **94.18%** attained by ResNet18-CNN, (9418 correct and 587 incorrect predictions) and **94.13%** by VGG16-CNN, (9413 correct and 587 incorrect predictions). The per-class distribution shows that SBNet-CNN has brought much fewer misclassifications to visually similar categories like the ones of Coat, Pullover and Shirt, in which traditional architectures have failed because of the overlapping feature boundaries. This has been enabled by the specific adaptive non-linearity added to the activation functions of the SMSA, which have added parameters of learnable slope and saturation ranges to each layer.

These dynamic characteristics have enabled SBNet-CNN to dynamically reconfigure activation responses to match the complexity of features in each layer thus improving intra-class compactness and inter-class separability. The power to refine non-linearity at a grain granular level has reinforced discriminative boundaries of ambiguous classes, resulting in a significant decrease in the misclassification errors. Consequently, SBNet-CNN has become even stronger in terms of both robustness and generalization as well as efficient feature encoding than the more complex ResNet18-CNN.

The ROC curves and the scores of the AUCs achieved on the test data with the SBNet-CNN architecture and ResNet18-CNN architecture fitted with the layer-wise adaptive SMSA activation function have been plotted on Fig 25 on the FashionMNIST dataset. The AUC value has measured the capability of the model to rank the classes and has captured the quality of features that are learned by the CNN as well as the performance of the activation function in modelling non-linear decision boundaries. SBNet-CNN using SMSA(x,β) has reached an AUC value of 1.00 on all ten classes, which implies flawless class separability. AUC values of 0.98 to 1.00 suggest extremely reliable discrimination by ResNet18-CNN with layer-wise SMSA across all classes. These findings on the test data have not only demonstrated the ability of the CNN architectures to produce discriminative features, but have also demonstrated the use of layer-wise adaptive activation in allowing each layer to tune its non-linearity to the complexity of representations at various depths. The high AUC scores achieved between the architectures have highlighted the efficiency of the SMSA mechanism to improve the decision boundaries even with minor inter-class similarities. Furthermore, the consistency of the findings in all classes has been used to show that the activation is highly generalizable despite training data. This layer adaptive has enabled the network to maintain high-ranking confidence with different complexities of features. These findings have shown that the proposed activation function can be scaled to architectures of different depth and representational hierarchies.

It has been suggested that the greater performance (**test accuracy 97.52%**) of SBNet-CNN in Table 22 than all other models was enabled by the fact that its architecture enabled the induction of compact yet expressive feature hierarchies through the strategic placement of convolutional blocks each with adaptive SMSA activations. The modulated gradient flow is using the learnable slope parameters of SMSA that accelerates convergence and removes vanishing gradients and adaptive range control that dynamically varies the sensitivity of activations to preserve discriminative features with varying data distributions. Further, the non-linearity ability of SMSA has facilitated each of the layers to offer autonomous balancing of sharp and smooth activations to offer a response that is explicitly adopted to the richness of the input features. This architecture conformity with lightweight CNN backbone and layer-wise adjustable non-linearity has been a great contributor to the ability of SBNet-CNN to produce high-level accuracy with reduced computational expense. SBNet-CNN has also been more adaptable to heterogeneous patterns of features compared to conventional CNNs which typically rely on fixed activation functions, i.e., ReLU or Swish. This has been particularly useful where there is a fine and context-specific inter-class variation. The network has not just done a better generalization of the undiscovered data but has also illustrated a better robustness in the challenging classification circumstances. One of the salient observations that are the result of the Fashion-MNIST experimental studies is succinctly summarized in Figs 7 and 11, Fig 21, where a Custom CNN with the addition of the mechanism of the SMSA(x, n) is shown and in Figs 7 and 11. SBNet with the addition of the mechanism of the adaptive activation SMSA(x,β) is demonstrated in Fig 21. Custom CNN structure, by definition, is relatively primitive and contains a limited number of trainable parameters; therefore, even though it can achieve a reasonable amount of feature generalization, it does not achieve complete convergence. On the other hand, the SBNet framework, which is combined with the adaptive activation, theSMSA(x,β) wisely uses non-linear transformation between consecutive layers. This adaptive modulation enables the network to develop more hierarchical representations which in turn spawn faster convergence, stabilised optimisation dynamics and greater levels of generalisation. Thus, SBNet is shown to be better at converging and more generalising compared to the less complex example of the Custom CNN. The deep networks such as ResNet18 and VGG16 can be constrained to their partially adaptive forms. In such models, multiple layers are fixed in place as a result of using pretrained weights that may limit gradient flowing and diminish complete end-to-end adaptiveness. However, low-level features are mostly learned at an early stage of the layers, whereas higher-to-lower levels generate more abstract and generalized features. Such mid-level representations can be improved by introducing layer-wise adaptive SMSA in about 70% of the trainable layers. This results in a marked improvement in the learning dynamics and classification performance as opposed to the case of the activated static settings. The analysis has included this observation and its implications.

**Table 22:** Performance comparison of the proposed SBNet-CNN model with existing architectures on the FashionMNIST dataset. Bold values indicate the proposed model.

Model [33]	Accuracy (%)
H-CNN model with VGG16	94.00
CNN-Softmax	91.86
LSTMs	88.26
CNN + HPO + Reg	93.99
CNNs	92.54
CNN LeNet-5	90.64
CNN	89.54
VGG	92.30
Shallow CNN	93.69
VGG Network	91.50
MCNN15	94.04
**Proposed SBNet-CNN**	**97.52**

#### Comparative evaluation of Optimized CNN and DenseNet121 against baseline CNNs for skin cancer and COVID-19 Classification.

**Fig 26:**
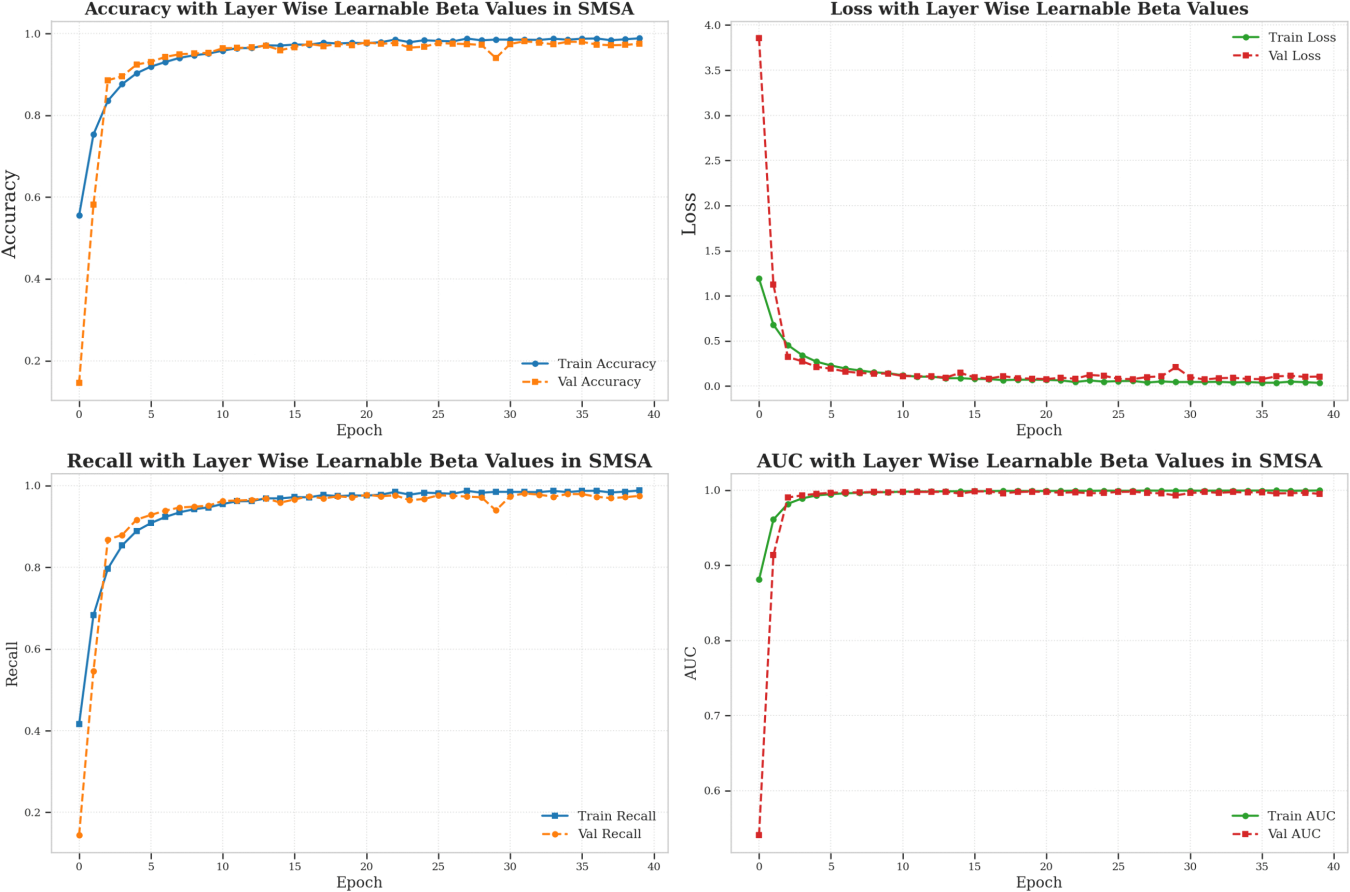
Effectiveness of layer-wise adaptive β modulation in SMSA for skin lesion detection.

**Fig 27:**
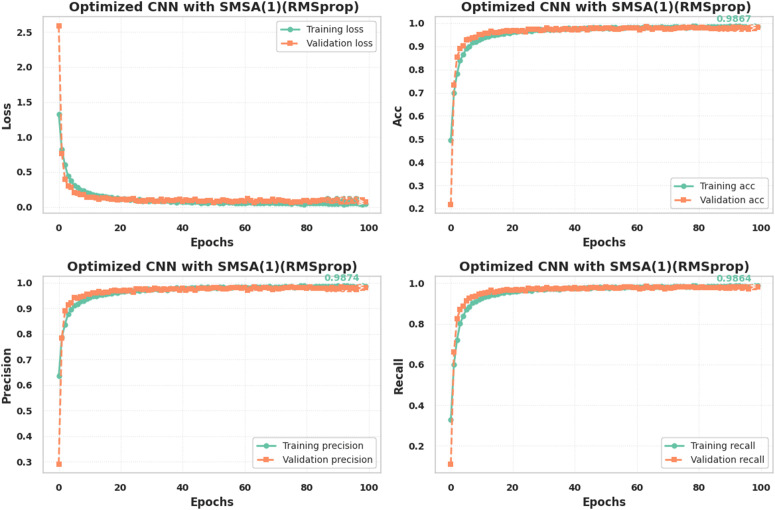
Accuracy and Loss Metric of Optimized CNN with SMSA(1) on HAM10000.

Convolutional neural networks have demonstrated very good performance Figs 26 and 27 in sensitive medical images like skin cancer classification. Adaptive activation functions have been incorporated in this performance to enhance it. The suggested SMSA model has brought in the learnable β parameters of each layer, which allows slope and curvature to be dynamically controlled. These parameters have enabled the network to be able to modify its nonlinearity in accordance to a layer-specific learning requirement. The development of the values of β in the various epochs in Fig 18 has proved convergence and interpretability. The layers have had a clear β curve, which represents how the network can be balanced to flexibly between expressiveness and regularization. This adaptivity has been able to increase shallow and deep extraction of features and this has improved robust learning. SMSA has been able to train faster compared with fixed activations without compromising or degrading classification accuracy. The model has recorded good performance on sensitive and complicated images of skin lesions. As can be seen, in Tables 23 and 24, the proposed Adaptive-SMSA is more predictive than Fixed SMSA(1) and training always. The increased training and validation accuracy (0.991 and 0.990) and very low loss values indicate that the adaptive model is more optimally stabilized and it has a better generalization. In addition to that, it also converges significantly faster (40 vs. 100 epochs) and reduces overall training time (224s vs. 500s), a fact that affirms the utility of learnable nonlinearity parameters in speeding up convergence and enhancing model achievement. These findings have established that adaptive activation functions are an important component to CNN generalization and robustness.

**Table 23:** Performance comparison between Adaptive β-SMSA and Fixed SMSA(1).

Metric	Adaptive β-SMSA	Fixed SMSA(1)
Train Accuracy	0.991	0.987
Validation Accuracy	0.990	0.9867
Train Loss	0.02	0.06
Validation Loss	0.03	0.06
Precision	0.990	0.9874
Recall	0.990	0.9864
AUC	0.995	Not shown
Generalization	Excellent	Excellent (slightly tighter)

**Table 24:** Training efficiency and nonlinearity control comparison of Adaptive β-SMSA and Fixed SMSA(1).

Aspect	Adaptive β-SMSA	Fixed SMSA(1)
Epochs to Converge	40	100
Time per Epoch (s)	5.6	5.0
Total Training Time (s)	**224**	**500**
Convergence Speed	Faster	Slower
Nonlinearity Control	Full (adaptive slope, range, concavity)	Limited (fixed *n*, slope only)
Form Flexibility	Layer-wise adaptive	Globally fixed shape
Computational Cost	Lower	Higher

and validation metrics.The class-wise performance presented in Table 25 shows that the Optimized CNN using SMSA(1) produces a very balanced and steady classification with all seven skin-lesion categories. The final test accuracy of 98.00% with a macro- and weighted F1-score of over 98 proves the strong multiclass discriminative power of the model. Some of the classes achieve a near-perfect accuracy and recall (such as Classes 0.0, 3.0, and 5.0), meaning acceptable identification with no significant false positives or false negatives. Even though Class 4.0 has a slightly lower recall at 89% it does not affect the overall macro and weighted measurements, thus confirming the presence of strong generalization despite the presence of class imbalance.

**Table 25:** Test classification performance metrics using SMSA(1) with Skin Cancer dataset.

Class	Precision (%)	Recall (%)	F1-Score (%)	Support
0.0	99.00	100.00	100.00	1359
1.0	99.00	100.00	99.00	1318
2.0	96.00	99.00	98.00	1262
3.0	100.00	100.00	100.00	1351
4.0	99.00	89.00	94.00	1374
5.0	100.00	100.00	100.00	1358
6.0	94.00	99.00	97.00	1365
**Accuracy**	98.00%			9387
**Macro Avg**	98.14	98.14	98.29	9387
**Weighted Avg**	98.13	98.00	98.10	9387

A direct comparison of the proposed Optimized CNN with the state-of-the-art methodologies is provided in Table 26, which makes it clear that the former is superior. Compared to antecedent approaches such as those proposed by Gupta, Shahin and Carcagni (accuracy is between 83.10% to 90.00%), and more complex architectures such as DCAN-Net (accuracy is 97.57%), the SMSA(1)-based CNN presented below achieves maximum accuracy (99.00% to 99.00%), highest precision (99.00%), recall (99.00%), F1-score (98.89%), and test accuracy (98.14%). This uniform improvement on all the measures of evaluation supports the augmented discriminative capacity bestowed by the SMSA activation mechanism.

**Table 26:** Comparison of Optimiz CNN with state-of-the-art methods on skin lesion classification tasks. Bold values indicate the proposed model.

Method	Precision	Recall	F1-score	Test Accuracy
Gupta [34]	89.00	83.00	83.00	83.10
Huang [35]	75.18	–	–	85.80
Shahin [36]	86.20	79.60	–	89.90
Carcagnì [37]	88.00	76.00	82.00	90.00
Chaturvedi [38]	88.00	88.00	–	93.20
Alsunaidi [39]	92.22	84.20	88.03	95.80
Aladhadh [40]	96.00	96.50	97.00	96.14
DCAN-Net [41]	97.00	97.57	97.10	97.57
**Optimiz CNN [SMSA(1)]**	**99.00**	**99.00**	**98.89**	**98.14**

Table 27 gives additional empirical data on the effectiveness of the Adaptive variant of the β SMSA used with the test dataset. Here, the Optimized CNN is better at all the other tested convolutional neural network architectures: precision of 98.45%, recall of 98.35%, F1-score of 98.49%, accuracy of 98.87%, and ROC-AUC of 99.40%. This study recorded a significantly high ROC-AUC score compared to the rest of the tested CNN architectures, such as MobileNet, ResNet-152v2, VGG variants, and U-Net based hybrids. This finding shows better separation of classes and threshold independent behavior, which further supports the usefulness of adaptive nonlinearity control. On the same note, Table 28 supports the long-term high performance of the proposed approach on the validation set. The model achieves a validation accuracy of 99.10% and ROC-AUC of 99.50% which is higher than all baseline architectures with a slight difference between validation and test values. The exceptionally small differences in Precision and F1-score also support the idea of the stability of learning dynamics and strong generalization. When combined, the results of all the tables support the main claims that the integration of SMSA, especially the Adaptive Beta SMSA, strengthens the stability of optimization, the increase in feature representation, and, finally, achieves better classification results compared to traditional CNN architectures.

**Table 27:** Test performance comparison of CNN-based models on skin lesion classification. Bold values indicate the proposed model.

Model	Precision (%)	Recall (%)	F1 Score (%)	Accuracy (%)	ROC-AUC (%)
MobileNet1 [42]	91.25	89.18	88.34	92.45	96.25
ResNet-152v2 [43]	90.34	88.57	87.65	92.57	95.32
VGG-16 [44]	90.78	89.27	88.95	92.89	94.21
MobileNet-V2 [45]	92.36	90.78	89.36	94.47	91.78
VGG-19 [46]	93.65	91.47	90.65	94.89	93.54
U-Net+MobileNet-V3 [47]	97.84	95.27	97.32	98.86	98.45
DenseNet-CNN [31]	68.80	70.59	69.68	93.24	90.00
**Optimized CNN 2**	**98.45**	**98.35**	**98.49**	**98.87**	**99.40**

**Table 28:** Validation performance comparison of CNN-based models on skin lesion classification. Bold values indicate the proposed model.

Model	Precision (%)	Recall (%)	F1 Score (%)	Accuracy (%)	ROC-AUC (%)
MobileNet1 [42]	90.18	89.03	87.89	92.02	95.89
ResNet-152v2 [43]	90.03	88.14	87.02	92.07	95.03
VGG-16 [44]	90.07	89.02	88.03	92.05	94.01
MobileNet-V2 [45]	91.98	90.01	89.01	94.03	91.07
VGG-19 [46]	93.01	90.09	88.04	94.08	93.02
U-Net+MobileNet-V3 [47]	97.32	94.07	97.03	98.03	97.09
**Optimized CNN 2**	**99.00**	**99.00**	**98.89**	**99.10**	**99.50**

The SMSA architecture captures the concept of implicit meta-learning and nonlinearity engineering through a parameter that is trainable, denoted β. That this innovation allows the simultaneous optimization of both synaptic weights and activation dynamics, it gives the model a layer-wise ability to modify its slope, curvature, and range of activation.The resultant adaptive modulation effectively counters gradient instability and at the same time increases the representational flexibility. Moreover, the learning-augmented activation paradigm is an exploration of nonlinear activation functions, where the exploration is nonlinear and continuously differentiable, thus avoiding reliance on heuristic nonlinearities. The hypothesis that more effective convergence is created by adaptive nonlinearity and increases the robustness of generalization in deep convolutional neural network models is supported by empirical evidence: higher classification accuracy, insignificant difference between validation and test performance, and significantly higher ROC-AUC values.

COVID-19 image classification has posed significant challenges for CNN-based models due to factors such as data scarcity, class imbalance, high inter-class similarity, heterogeneous imaging sources, and the presence of subtle pathological features. To mitigate these issues, a DenseNet-121 model integrated with the adaptive SMSA activation function has been employed. The proposed model has achieved a classification accuracy of **99.03%**, an AUC of **98.74%**, and a precision of **98.2%**, as presented in Table 29. Compared to standard DenseNet-121 in Table 30, which achieved 97.03% accuracy, the inclusion of the SMSA activation has improved performance by approximately 2%. Furthermore, training time has been reduced from 989 seconds to 380 seconds, demonstrating both computational efficiency and improved learning capability. The Table 31 has shown that the proposed model also outperformed several state-of-the-art methods from the literature in terms of sensitivity, AUC, and overall classification accuracy.

**Table 29:** Final training and validation accuracy/loss of DenseNet CNN with adaptive β-SMSA activation on COVID-19 dataset.

Metric	Train	Validation	Best Epoch
Accuracy	0.988	0.990	20
Loss	0.043	0.040	20

**Table 30:** A comparison of the proposed model’s metrics with those of other individual networks. Bold values indicated proposed model.

Model	Sensitivity	Specificity	Precision	AUC	Accuracy	Training Time (secs)
ResNet-50 [48]	1	1	0.83	93.27	82.20	1123
VGG-16 [48]	0.9	0.9	0.94	92.6	94.22	1237
MobileNetV2 [48]	0.98	0.98	0.96	98.88	96.36	1336
DenseNet-121 [48]	0.99	0.99	0.97	96.34	97.03	989
**DenseNet-121 (SMSA 2)**	0.99	0.99	0.982	98.74	99.03	380

**Table 31:** A comparison of the proposed model with baseline models. Bold values indicated proposed model.

Literature Rewiew	Citation	Sensitivity	Precision	AUC	Accuracy	Training Time (Secs)
K. Wang et al.	[5]	97.76	92.6	95.21	92.8	1012
Sivaramakrishnan et al.	[49]	–	97.7	99.3	96.2	1079
Farhan Sadik et al.	[50]	32.4	–	–	65.7	1135
Adhiyaman et al.	[51]	–	88.97	–	93.06	1232
Chouhan et al.	[52]	99.62	93.28	99.34	96.39	1346
Enes Ayan et al.	[53]	89.1	91.3	87	84.5	1354
DenseNet-121	[48]	99	87.70	96.34	97.03	989
**DenseNet-121 (SMSA 2)**	(Proposed)	99.0	99.0	98.20	98.74	380

**Fig 28:**
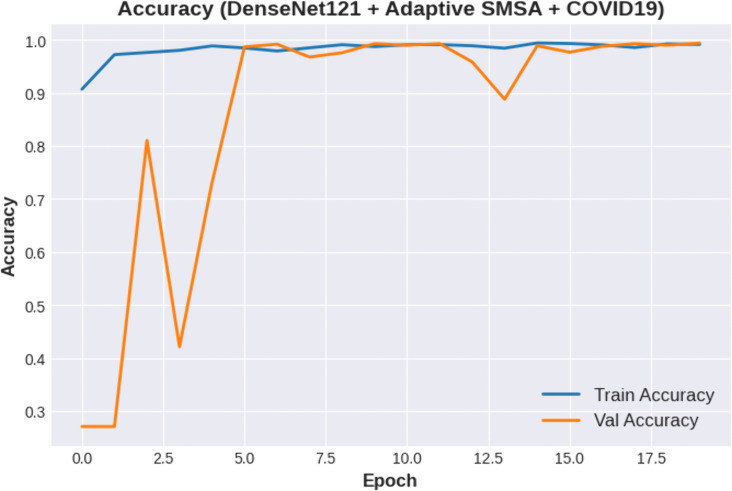
Performance Metric Adaptive β of SMSA in DenseNet121 CNN for COVID-19.

**Fig 29:**
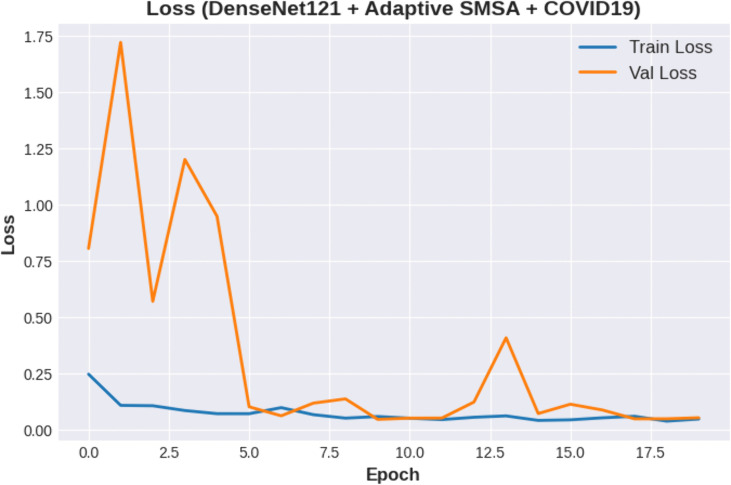
Performance Metric Adaptive β of SMSA in DenseNet121 CNN for COVID-19.

**Fig 30:**
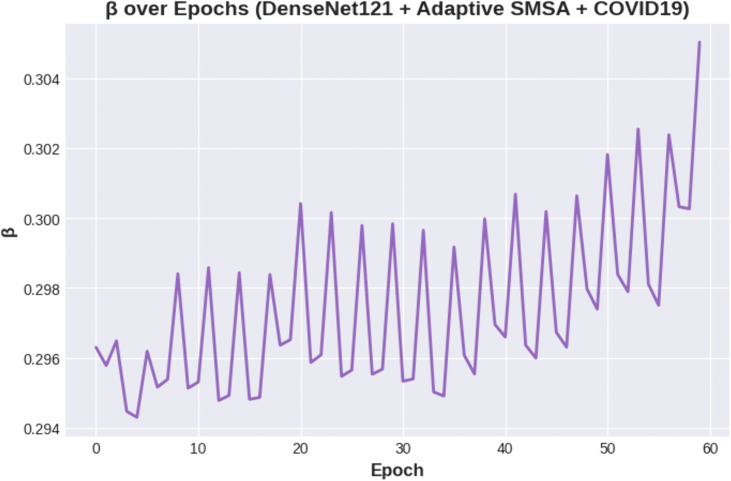
Learnable β trends indicating fine-tuning of nonlinearity in Adaptive SMSA during training (DenseNet121, COVID-19).

The Figs presented above, Figs 28 and 29, show the training and validation accuracy/loss curves. The training accuracy has also been at an average of over 98.5% across all the epochs although the validation accuracy has been more than 99% after the 10th epoch. The loss on validation has drastically reduced after epoch 5 and stayed constant at small values, reflecting a high degree of generalisation to unseen data and no overfitting, despite a small and imbalanced dataset. SMSA adaptiveness of the activation functionality has been a main contributing factor to this enhancement. As shown in Fig 30. The trainable β coefficient has been steadily growing between about 0.295 and 0.304 as it has been trained.

This adaptive response has seen the network dynamically adapt its non-linear response to model more subtle, diffused manifestations of COVID-19 including ground-glass opacities and consolidations. The DenseNet-121 model with SMSA activation has shown to be superior in COVID-19 classification due to its improved capability to perform feature extraction, overfitting control, and dynamic non-linearity. Computational efficiency and generalizability of the model viewpoint indicate that the model can be applied in real-time clinical settings. Combined, the two versions of SMSA have done image classification problems more and more efficiently as the nonlinear representational requirements of the deep convolutional neural network designs. Activation Learning slopes and curvatures may be adaptively rescaled using a learnable hyperparameter whose name is the β, thereby making an implicit kind of meta-learning, the transformation dynamics as well as network weights are co-optimized. These nonlinearity which are designed assist to enhance discrimination feature learning, stabilize gradient propagation and decrease the gap between the validation and the test performance. Empirical evidence in the current research demonstrates the concept that the SMSA framework can not only offer optimum classification and classification but also guarantee sound reliable, generalization of image information sets of various and complex data.

### Statistical validation of classification performance: paired t-test and confidence interval analysis for ResNet18 and SBNet CNN architectures

As summarized in Table 32 represents the statistical analysis that has provided useful insights into the performance of the ResNet18 CNN and SBNet CNN models. The 95% confidence intervals, namely [0.915, 0.945] in the case of ResNet18 and [0.968, 0.972] in the case of SBNet, suggest that the accuracy of the classification estimates are very precise. The mean accuracy of the SBNet-CNN model is greater thus indicating a high classification ability as compared to ResNet18. The very high t-values of ResNet18 and SBNet 108.47 and 852.94 respectively with a p-value of less than 0.0001, provide strong statistical evidence against the null hypothesis that the baseline accuracy equals 0.10. These results support the fact that both models have significantly improved their performance. What is more, the low standard error of the values indicates the low level of variation of the values among the folds, which further increases the confidence in the generalization and reliability of the model

**Table 32:** Comprehensive statistical analysis of ResNet18-CNN and SBNet-CNN using confidence levels, confidence intervals, t-tests, and significance evaluation.

Model	Mean	CI (95%)	SE	Confidence Level	t-Value	p-Value	Significance
**ResNet18-CNN**	0.930	[0.915, 0.945]	0.007653	95%	108.47	$2.62\times 10^{-13}$	**Comparative**
**SBNet-CNN**	0.970	[0.968, 0.972]	0.001020	95%	852.94	$4.23\times 10^{-22}$	**Superlative**

**Fig 31:**
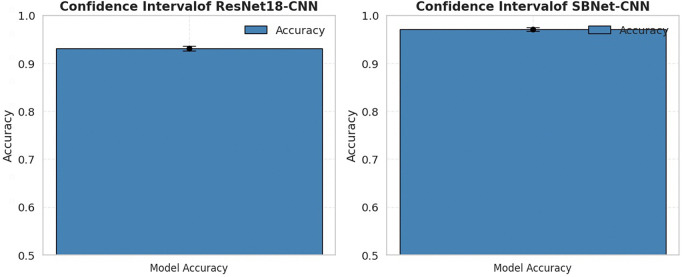
Comparison of classification accuracy between ResNet18-CNN and SBNet-CNN models across test experimental conditions.

**Fig 32:**
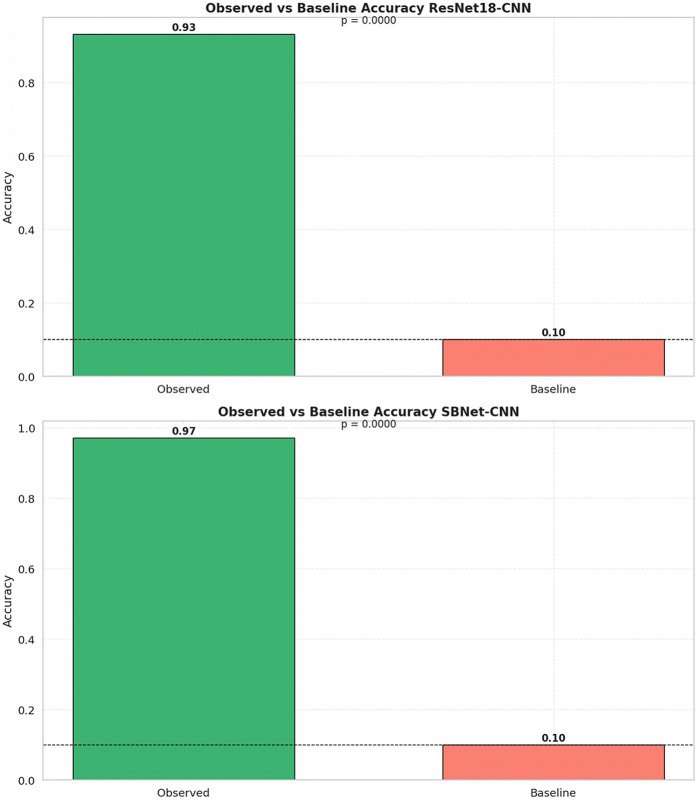
Baseline versus observed mean accuracies for ResNet18-CNN and SBNet-CNN, including error margins.

Fig 31 visually shows the relative accuracy of classification of the ResNet18 and SBNet CNNs, and Fig 32 uses results to show the difference between the observed mean accuracy and the expected value of the accuracy, and the error margins. Combined, these analyses prove the suitability of the two networks, with a significantly better and more consistent performance of SBNet-CNN on the Fashion MNIST dataset.

## Conclusion

This paper addresses the question of how to design adaptable nonlinear activation functions in a convolutional neural network by proposing Symmetric Modifiable Slope Activation (SMSA) function and analyzing them in three different variants: manually tuned SMSA with a fixed slope parameter n, globally adaptive SMSA with a learnable slope parameter β, and the proposed layer-wise adaptive SMSA where SMSA has a learnable slope parameter, β, independently learned through each convolutional layer, in its three variants which are described by Eq 2. Systematic evaluation was conducted on datasets of different structural complexity, which included MNIST, Fashion MNIST, HAM10000, and COVID −19, using small and large CNNs, namely Custom CNN, SBNet -CNN, ResNet18, VGG16, Optimized CNN, and DenseNet121. Experimental findings indicate that the manually tuned SMSA has good feature discrimination with the slope parameter n chosen according to the characteristics of the dataset. However, the above method presents the reliance on manually-acquired hyperparameters, which limits its ability to scale-up to heterogeneous tasks. This problem is solved by globally adaptive-SMSA, which does not change the nonlinearity of the network, but independently optimizes the slope of activation by the gradient descent mechanism. The greatest improvement is achieved in the form of the proposed layer-wise adaptive SMSA, which allows every convolutional layer to employ its own nonlinear transformation. This is an adaptive process that inhibits divergence, regularizes the gradient propagation and also enables hierarchical learning of salient features. The effectiveness of the suggested method is supported by empirical assessments. The SBNet-CNN with layer-wise adaptive SMSA achieved a test, validation and training accuracy of 97.52%, 98.89% and 99.1% respectively on the Fashion-MNIST dataset and only took 17 training epochs to converge, in comparison to more complex networks like ResNet-18 (94.18%) and VGG-16 (94.13%). In a similar manner, experiments run on the HAM10000 data set exhibited an improvement in the feature discriminability and faster convergence. Besides, when integrated with DenseNet-121 on the COVID-19 dataset, integration demonstrated a higher robustness considering extreme imbalance in classes and was about 4% more precise compared to the baseline model. On the whole, these results emphasize the importance of the adaptability of activations to deep learning system optimization. The proposed layer-wise adaptive-SMSA allows nonlinear transformations to be heterogeneous across network depth, allowing each layer to work in a favorable activation regime. This method is therefore significantly more stable in optimization, higher in gradient propagation, and able to cross-dataset (and cross-architecture) generalize. Further developments in the use of SMSA in transformer-based models, graph neural networks, and large-scale vision systems and theoretical investigations into the dynamics of adaptive activation curvature should be explored in future research to better understand the role of nonlinear adaptability in deep representation learning.
