# Supplementary material for: Development of adaptive activation functions with curvature and range modulation for abstract image feature learning in complex CNNs for cross-domain applications
Source: PLoS One. 2026 Aug 14;21(8):e0355613. doi: 10.1371/journal.pone.0355613 (PMC13475904; doi:10.1371/journal.pone.0355613)
Supplement: Supplementary file 1 [file pone.0355613.s002.docx]

# Response to Reviewers

Manuscript ID: PONE-D-25-53406

Title: Development of Adaptive Activation Functions with Curvature and Range Modulation for Abstract Image Feature Learning in Complex CNNs for Cross-Domain Applications

Dear Editor and Reviewer,

We sincerely thank the editor and reviewer for their constructive comments and valuable suggestions. The manuscript has been carefully revised to address all concerns. All corrections have been incorporated in the revised manuscript.

**Responses to Editorial Comments**

- **Comment:** Please ensure that your manuscript meets PLOS ONE's style requirements, including those for file naming. The PLOS ONE style templates can be found [https://journals.plos.org/plosone/s/file?id=wjVg/PLOSOne_formatting_sample_main_body.pdf](https://journals.plos.org/plosone/s/file?id=wjVg/PLOSOne_formatting_sample_main_body.pdf), [https://journals.plos.org/plosone/s/file?id=ba62/PLOSOne_formatting_sample_title_authors_affiliations.pdf](https://track.editorialmanager.com/CL0/https:%2F%2Fjournals.plos.org%2Fplosone%2Fs%2Ffile%3Fid=ba62%2FPLOSOne_formatting_sample_title_authors_affiliations.pdf/1/010f019c4ffd6d32-7945d2f8-dd46-49be-8e9c-77018c3b4664-000000/HgZjICsmmnuk7xWdlNVKoyXkIpqY4SYGSM8TZQxB2bY=250)

**Response:** The manuscript has been revised according to the journal formatting requirements, including figure captions, table references, and manuscript structure.

- We note that some of your figures are in png format and are not displayed correctly in the compiled file for review. Please check that your figures are visible when submitting your revised version. If you have any issues please contact the journal office at [plosone@plos.org](mailto:plosone@plos.org) for assistance.

**Response:** All figures have been replaced with high-resolution versions and verified to ensure correct display in the compiled manuscript.

- Please note that PLOS One has specific guidelines on code sharing for submissions in which author-generated code underpins the findings in the manuscript. In these cases, we expect all author-generated code to be made available without restrictions upon publication of the work. Please review our guidelines at [https://journals.plos.org/plosone/s/materials-and-software-sharing#loc-sharing-code](https://journals.plos.org/plosone/s/materials-and-software-sharing#loc-sharing-code) and ensure that your code is shared in a way that follows best practice and facilitates reproducibility and reuse.

**Response:** The implementation code used for the proposed activation function and experiments will be made publicly available in an online repository to ensure reproducibility. All data required to reproduce the results, including values used in graphs and performance tables, are included within the manuscript <https://github.com/Aliraza927/Aliraza/blob/984cd2301c2fc3b0e7d7f49df4c2357dd220467c/SMSA1_WORK.ipynb>

- Please ensure that you refer to Figure 3 and 4 in your text as, if accepted, production will need this reference to lin the reader to the figure.

**Response** All supplementary materials have been relabeled as Supporting Information, and legends have been added after the references section.

- We note you have included a table to which you do not refer in the text of your manuscript. Please ensure that you refer to Table 6, 7, 8, 9, 17, 22, 23, 24, 25, 29 and 30 in your text; if accepted, production will need this reference to link the reader to the Table.

**Response:** We have carefully reviewed the manuscript and ensured that all tables, including Tables 6, 7, 8, 9, 17, 22, 23, 24, 25, 29, and 30, are now appropriately cited and discussed within the text. This allows readers to easily locate and interpret the information presented in each table.

**Responses to Reviewer Comments**

- **Scalability and generalization of manual tuning:** SMSA (x, n) depends on dataset-specific manual tuning of the slope parameter n. This methodology does not scale effectively to new tasks. It increases the engineering workload, as evidenced by the authors’ observation that manual tuning improves performance but does not generalize across tasks. Please comment on this assumption.

**Response:** We agree with the reviewer’s observation that the manually tuned formulation SMSA(x, n) introduces additional engineering effort and may not scale effectively across diverse tasks. The manually tuned form was primarily introduced to analyze the curvature behavior and interval-dependent gradient characteristics of the proposed activation function. To address this limitation, adaptive variants SMSA(x, β) and layer-wise learnable parameters were introduced, enabling automatic parameter optimization during training and thereby improving scalability and generalization across datasets and architectures. This clarification has been incorporated into the **Discussion section** of the revised manuscript.

- **Incomplete end-to-end adaptivity in some architectures:** In deeper neural networks such as ResNet18 and VGG16, only a subset of the layers was made adaptive (for instance, approximately 70% of ResNet18 layers with adaptive SMSA), while the remaining pretrained layers remained fixed. This partial adaptivity may result in constraints on gradient flow and, in certain instances, cause deterioration of gradient propagation within the non-adapted sections, thereby restricting potential improvements. Please comment on this idea.

**Response:** We agree with the reviewer’s observation regarding the potential limitations of partial adaptivity in deeper architectures such as ResNet18 and VGG16. In these models, several layers remained fixed due to the use of pretrained weights, which may impose certain constraints on gradient propagation and can negatively affect full end-to-end adaptivity. However, early layers primarily learn low-level features, while mid-to-lower layers capture more abstract and generalized representations; introducing layer-wise adaptive SMSA in approximately 70% of the trainable layers effectively enhances these mid-level representations. As a result, learning dynamics and classification performance improved significantly compared with static activation settings. This clarification and its implications have been incorporated into the **Discussion section** of the revised manuscript submitted.

- **Dependency on dataset characteristics:** The documented enhancements are notably evident in cross-domain and imbalanced data scenarios (e.g., HAM10000, COVID-19). It remains uncertain to what extent significant improvements are observed on conventional, balanced benchmarks, indicating that the method’s benefits might be predominantly domain-specific and may not consistently extend to all datasets without meticulous adjustment. What is the opinion of the authors about this dependency?

**Response:** We agree with the reviewer’s observation regarding the possible dataset dependency of the proposed method. While the improvements are more pronounced on challenging and imbalanced datasets such as HAM10000 and COVID-19, experiments on balanced benchmarks including MNIST and Fashion-MNIST also demonstrate stable and competitive performance. These observations suggest that SMSA is not strictly domain-specific, although its advantages become more visible under complex data distributions. A broader evaluation across additional domains will be considered in future research to further examine its generalization capability.

- **Potential risk of overfitting with added flexibility**: The introduction of per-layer β parameters enhances the model's capacity. Although the paper reports improved generalization in their experiments, the additional degrees of freedom may pose a risk of overfitting in regimes with limited data or when not accompanied by appropriate regularization and validation. Please discuss this risk.

**Response:** We agree with the reviewer that introducing layer-wise learnable β parameters may increase the risk of overfitting due to the added flexibility. However, in our formulation the number of additional parameters remains very small, as the number of β parameters is equal only to the number of layers. Empirical results indicate that this limited increase in parameters improves the rate of convergence, overall convergence behavior, and generalization of the CNN compared with static activation functions. The only trade-off observed is a slight increase in computational cost, with marginally higher GPU time per epoch, which has now been clarified in the **Discussion section** of the revised manuscript submitted.

- The text also could need improvement. It contains a few writing errors and typos, some of which are listed below.
  1. complex non-linearity aproximation function ; Gradient modifiability Of SMSA; (see Fig.( 11)) ; evidenced in Fig.( ??). ; 0.36 obserserved in ; ….
  2. Most of the figures need improvement. Some present blurring effects, and some have typos. Please correct them.

**Response:** We thank the reviewer for carefully identifying the writing errors and typographical issues in the manuscript. All highlighted terms (e.g., “aproximation,” “obserserved,” figure references, and formatting inconsistencies) have been carefully corrected throughout the revised manuscript. In addition, several figures have been improved by enhancing their resolution and correcting typographical errors to ensure better clarity and readability. These revisions have been implemented across the entire manuscript submitted
