# Supplementary material for: Development of adaptive activation functions with curvature and range modulation for abstract image feature learning in complex CNNs for cross-domain applications
Source: PLoS One. 2026 Aug 14;21(8):e0355613. doi: 10.1371/journal.pone.0355613 (PMC13475904; doi:10.1371/journal.pone.0355613)
Supplement: Peer review history

# Peer review: Decision Letter 0

**DOI:** 10.1371/journal.pone.0355613.r001

**Authors:** Joanna Tindall

11 Feb 2026

Dear Dr. Ali,

Please submit your revised manuscript by Mar 26 2026 11:59PM. If you will need more time than this to complete your revisions, please reply to this message or contact the journal office at plosone@plos.org. When you're ready to submit your revision, log on to https://www.editorialmanager.com/pone/ and select the 'Submissions Needing Revision' folder to locate your manuscript file.

- A letter that responds to each point raised by the academic editor and reviewer(s). You should upload this letter as a separate file labeled 'Response to Reviewers'.
- A marked-up copy of your manuscript that highlights changes made to the original version. You should upload this as a separate file labeled 'Revised Manuscript with Track Changes'.
- An unmarked version of your revised paper without tracked changes. You should upload this as a separate file labeled 'Manuscript'.

If you would like to make changes to your financial disclosure, please include your updated statement in your cover letter. Guidelines for resubmitting your figure files are available below the reviewer comments at the end of this letter.

If applicable, we recommend that you deposit your laboratory protocols in protocols.io to enhance the reproducibility of your results. Protocols.io assigns your protocol its own identifier (DOI) so that it can be cited independently in the future. For instructions see: https://journals.plos.org/plosone/s/submission-guidelines#loc-laboratory-protocols. Additionally, PLOS ONE offers an option for publishing peer-reviewed Lab Protocol articles, which describe protocols hosted on protocols.io. Read more information on sharing protocols at https://plos.org/protocols?utm_medium=editorial-email&utm_source=authorletters&utm_campaign=protocols.

We look forward to receiving your revised manuscript.

Kind regards,

Joanna Tindall, PhD

Staff Editor

PLOS One

Journal Requirements:

When submitting your revision, we need you to address these additional requirements.

1.Please ensure that your manuscript meets PLOS ONE's style requirements, including those for file naming. The PLOS ONE style templates can be found at

https://journals.plos.org/plosone/s/file?id=wjVg/PLOSOne_formatting_sample_main_body.pdf and

https://journals.plos.org/plosone/s/file?id=ba62/PLOSOne_formatting_sample_title_authors_affiliations.pdf

2. We note that some of your figures are in png format and are not displayed correctly in the compiled file for review. Please check that your figures are visible when submitting your revised version. If you have any issues please contact the journal office at plosone@plos.org for assistance.

3. Please note that PLOS One has specific guidelines on code sharing for submissions in which author-generated code underpins the findings in the manuscript. In these cases, we expect all author-generated code to be made available without restrictions upon publication of the work. Please review our guidelines at https://journals.plos.org/plosone/s/materials-and-software-sharing#loc-sharing-code and ensure that your code is shared in a way that follows best practice and facilitates reproducibility and reuse.

4. We notice that your supplementary figures are uploaded with the file type 'Figure'. Please amend the file type to 'Supporting Information'. Please ensure that each Supporting Information file has a legend listed in the manuscript after the references list.

5. We note that your Data Availability Statement is currently as follows: “All relevant data are within the manuscript and its Supporting Information files.”

Please confirm at this time whether or not your submission contains all raw data required to replicate the results of your study. Authors must share the “minimal data set” for their submission. PLOS defines the minimal data set to consist of the data required to replicate all study findings reported in the article, as well as related metadata and methods (https://journals.plos.org/plosone/s/data-availability#loc-minimal-data-set-definition).

For example, authors should submit the following data:

- The values behind the means, standard deviations and other measures reported;

- The values used to build graphs;

- The points extracted from images for analysis.

Authors do not need to submit their entire data set if only a portion of the data was used in the reported study.

If your submission does not contain these data, please either upload them as Supporting Information files or deposit them to a stable, public repository and provide us with the relevant URLs, DOIs, or accession numbers. For a list of recommended repositories, please see https://journals.plos.org/plosone/s/recommended-repositories.

If there are ethical or legal restrictions on sharing a de-identified data set, please explain them in detail (e.g., data contain potentially sensitive information, data are owned by a third-party organization, etc.) and who has imposed them (e.g., an ethics committee). Please also provide contact information for a data access committee, ethics committee, or other institutional body to which data requests may be sent. If data are owned by a third party, please indicate how others may request data access.

6. Please ensure that you refer to Figure 3 and 4 in your text as, if accepted, production will need this reference to link the reader to the figure.

7. We note you have included a table to which you do not refer in the text of your manuscript. Please ensure that you refer to Table 6, 7, 8, 9, 17, 22, 23, 24, 25, 29 and 30 in your text; if accepted, production will need this reference to link the reader to the Table.

8. If the reviewer comments include a recommendation to cite specific previously published works, please review and evaluate these publications to determine whether they are relevant and should be cited. There is no requirement to cite these works unless the editor has indicated otherwise.

Additional Editor Comments:

Please find the reviewer comments below. Please note that we have only been able to secure a single reviewer to assess your manuscript. We are issuing a decision on your manuscript at this point to prevent further delays in the evaluation of your manuscript. Please be aware that the editor who handles your revised manuscript might find it necessary to invite additional reviewers to assess this work once the revised manuscript is submitted. However, we will aim to proceed on the basis of this single review if possible.

[Note: HTML markup is below. Please do not edit.]

Reviewer's Responses to Questions

**Comments to the Author**

1. Is the manuscript technically sound, and do the data support the conclusions?

Reviewer #1: Yes

**********

2. Has the statistical analysis been performed appropriately and rigorously?

Reviewer #1: Yes

**********

3. Have the authors made all data underlying the findings in their manuscript fully available?

The PLOS Data policy

Reviewer #1: Yes

**********

4. Is the manuscript presented in an intelligible fashion and written in standard English?

Reviewer #1: Yes

**********

Reviewer #1: Review of article: PONE-D-25-53406

“Development of Adaptive Activation Functions with Curvature and Range Modulation for Abstract Image Feature Learning in Complex CNNs for Cross-Domain Applications “

The article introduces a novel Adaptive Symmetric Modifiable Slope Activation function (SMSA) designed for convolutional neural networks (CNNs). SMSA adjusts the curvature and range of activations to enhance the learning of abstract image features. Additionally, SMSA stabilizes gradient flow by incorporating near-zero convexity and controlled concavity for larger inputs, thereby mitigating issues related to vanishing and exploding gradients. This method demonstrates notable improvements in cross-domain applications, such as HAM10000 and COVID-19 datasets, particularly under conditions of data imbalance, with enhanced generalization capabilities. When compared to fixed activation functions such as ReLU, Leaky ReLU, ELU, Swish, and Mish, models employing SMSA frequently achieve superior metrics—including accuracy and ROC-AUC—faster convergence, and greater parameter efficiency.

The results presented in the article are promising; however, I recommend the following points to improve the article:

• Scalability and generalization of manual tuning

o SMSA (x, n) depends on dataset-specific manual tuning of the slope parameter n. This methodology does not scale effectively to new tasks. It increases the engineering workload, as evidenced by the authors’ observation that manual tuning improves performance but does not generalize across tasks. Please comment on this assumption.

• Incomplete end-to-end adaptivity in some architectures

o In deeper neural networks such as ResNet18 and VGG16, only a subset of the layers was made adaptive (for instance, approximately 70% of ResNet18 layers with adaptive SMSA), while the remaining pretrained layers remained fixed. This partial adaptivity may result in constraints on gradient flow and, in certain instances, cause deterioration of gradient propagation within the non-adapted sections, thereby restricting potential improvements. Please comment on this idea

• Dependency on dataset characteristics

o The documented enhancements are notably evident in cross-domain and imbalanced data scenarios (e.g., HAM10000, COVID-19). It remains uncertain to what extent significant improvements are observed on conventional, balanced benchmarks, indicating that the method’s benefits might be predominantly domain-specific and may not consistently extend to all datasets without meticulous adjustment. What is the opinion of the authors about this dependency?

• Potential risk of overfitting with added flexibility

o The introduction of per-layer β parameters enhances the model's capacity. Although the paper reports improved generalization in their experiments, the additional degrees of freedom may pose a risk of overfitting in regimes with limited data or when not accompanied by appropriate regularization and validation. Please discuss this risk.

The text also could need improvement. It contains a few writing errors and typos, some of which are listed below.

complex non-linearity aproximation function ; Gradient modifiability Of SMSA; (see Fig.( 11)) ; evidenced in Fig.( ??). ; 0.36 obserserved in ; ….

Most of the figures need improvement. Some present blurring effects, and some have typos. Please correct them.

**********

what does this mean?). If published, this will include your full peer review and any attached files.

If you choose “no”, your identity will remain anonymous but your review may still be made public.

**Do you want your identity to be public for this peer review?**  For information about this choice, including consent withdrawal, please see our Privacy Policy

Reviewer #1: **Yes:**  Leopoldo Altamirano Robles

**********

[NOTE: If reviewer comments were submitted as an attachment file, they will be attached to this email and accessible via the submission site. Please log into your account, locate the manuscript record, and check for the action link "View Attachments". If this link does not appear, there are no attachment files.]

To ensure your figures meet our technical requirements, please review our figure guidelines: https://journals.plos.org/plosone/s/figures

You may also use PLOS’s free figure tool, NAAS, to help you prepare publication quality figures: https://journals.plos.org/plosone/s/figures#loc-tools-for-figure-preparation.

NAAS will assess whether your figures meet our technical requirements by comparing each figure against our figure specifications.

# Author comment: Author response to Decision Letter 1

**DOI:** 10.1371/journal.pone.0355613.r002

6 Apr 2026

Dear Reviewers,

We are grateful to the reviewers for the insightful comments and suggestions, which have helped us significantly improve the quality and clarity of the manuscript.

We have carefully addressed all reviewer comments and revised the manuscript accordingly. A detailed, point-by-point response to each comment has been provided in the response-to-reviewers document. Changes made in the manuscript have been highlighted for ease of review.

Attachment Submitted filename: Response to Reviewers.docx

# Peer review: Decision Letter 1

**DOI:** 10.1371/journal.pone.0355613.r003

**Authors:** Zahid Mehmood

24 Jul 2026

Development of Adaptive Activation Functions with Curvature and Range Modulation for Abstract Image Feature Learning in Complex CNNs for Cross-Domain Applications

PONE-D-25-53406R1

Dear Dr. Ali,

We’re pleased to inform you that your manuscript has been judged scientifically suitable for publication and will be formally accepted for publication once it meets all outstanding technical requirements.

Within one week, you’ll receive an e-mail detailing the required amendments. When these have been addressed, you’ll receive a formal acceptance letter and your manuscript will be scheduled for publication.

An invoice will be generated when your article is formally accepted. Please note, if your institution has a publishing partnership with PLOS and your article meets the relevant criteria, all or part of your publication costs will be covered. Please make sure your user information is up-to-date by logging into Editorial Manager at Editorial Manager® and clicking the ‘Update My Information' link at the top of the page. For questions related to billing, please contact billing support.

If your institution or institutions have a press office, please notify them about your upcoming paper to help maximize its impact. If they’ll be preparing press materials, please inform our press team as soon as possible -- no later than 48 hours after receiving the formal acceptance. Your manuscript will remain under strict press embargo until 2 pm Eastern Time on the date of publication. For more information, please contact onepress@plos.org.

Kind regards,

Zahid Mehmood, PhD

Academic Editor

PLOS One

Additional Editor Comments (optional):

Reviewers' comments:

Reviewer's Responses to Questions

**Comments to the Author**

Reviewer #1: All comments have been addressed

Reviewer #2: All comments have been addressed

**********

2. Is the manuscript technically sound, and do the data support the conclusions?

Reviewer #1: Yes

Reviewer #2: Yes

**********

3. Has the statistical analysis been performed appropriately and rigorously?

Reviewer #1: Yes

Reviewer #2: Yes

**********

4. Have the authors made all data underlying the findings in their manuscript fully available?

The PLOS Data policy

Reviewer #1: Yes

Reviewer #2: Yes

**********

5. Is the manuscript presented in an intelligible fashion and written in standard English?

Reviewer #1: Yes

Reviewer #2: Yes

**********

Reviewer #1: No comments. All my comments that I have rised in the previous round habe been revised, so I dont have any new comments.

Reviewer #2: The paper is very good. I can’t review such this excellent paper. It should be accepted and published directly without anything. Congratulations!

**********

what does this mean?). If published, this will include your full peer review and any attached files.

If you choose “no”, your identity will remain anonymous but your review may still be made public.

**Do you want your identity to be public for this peer review?**  For information about this choice, including consent withdrawal, please see our Privacy Policy

Reviewer #1: **Yes:**  Leopoldo Altamirano Robles

Reviewer #2: No

**********

# Editor report: Acceptance letter

**DOI:** 10.1371/journal.pone.0355613.r004

**Authors:** Zahid Mehmood

PONE-D-25-53406R1

PLOS One

Dear Dr. Ali,

I'm pleased to inform you that your manuscript has been deemed suitable for publication in PLOS One. Congratulations! Your manuscript is now being handed over to our production team.

At this stage, our production department will prepare your paper for publication. This includes ensuring the following:

* All references, tables, and figures are properly cited

* All relevant supporting information is included in the manuscript submission,

* There are no issues that prevent the paper from being properly typeset

You will receive further instructions from the production team, including instructions on how to review your proof when it is ready. Please keep in mind that we are working through a large volume of accepted articles, so please give us a few days to review your paper and let you know the next and final steps.

Lastly, if your institution or institutions have a press office, please let them know about your upcoming paper now to help maximize its impact. If they'll be preparing press materials, please inform our press team within the next 48 hours. Your manuscript will remain under strict press embargo until 2 pm Eastern Time on the date of publication. For more information, please contact onepress@plos.org.

You will receive an invoice from PLOS for your publication fee after your manuscript has reached the completed accept phase. If you receive an email requesting payment before acceptance or for any other service, this may be a phishing scheme. Learn how to identify phishing emails and protect your accounts at https://explore.plos.org/phishing.

If we can help with anything else, please email us at customercare@plos.org.

Thank you for submitting your work to PLOS ONE and supporting open access.

Kind regards,

PLOS ONE Editorial Office Staff

on behalf of

Dr. Zahid Mehmood

Academic Editor

PLOS One
